# Carbon-Promoted Pt-Single Atoms Anchored on RuO_2_ Nanorods to Boost Electrochemical Hydrogen Evolution

**DOI:** 10.1021/acsami.4c06033

**Published:** 2024-05-17

**Authors:** Jing-Fang Huang, Wen-Jun Hsieh, Jeng-Lung Chen

**Affiliations:** † A Department of Chemistry, National Chung Hsing University, Taichung 402, Taiwan (R.O.C); ‡ National Synchrotron Radiation Research Center, Science-Based Industrial Park, Hsinchu30076, Taiwan (R.O.C)

**Keywords:** single-atom catalysts, RuO_2_ nanorods, Pt, hydrogen evolution reaction, water splitting

## Abstract

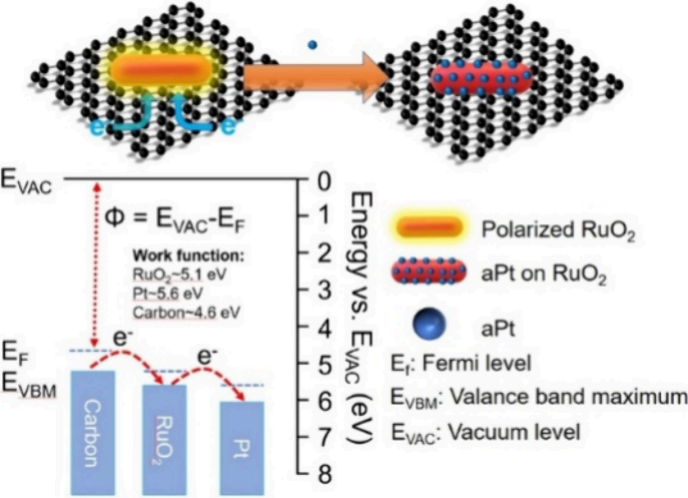

While efficient for electrochemical hydrogen evolution reaction (HER), Pt is limited by its cost and rarity. Traditional Pt catalysts and Pt single-atom (aPt) catalysts (Pt-SACs) face challenges in maintaining kinetically favorable HER pathways (Volmer–Tafel) at ultralow Pt loadings. Herein, carbon-promoted aPts were deposited on RuO_2_ without the addition of reductants. aPts confined on carbon-supported RuO_2_ nanorods (aPt/RuO_2NR_/Carbon) promoted “inter-aPts” Tafel. aPt/RuO_2NR_/Carbon is the Pt-SAC that retained underpotentially deposited H; additionally, its HER onset overpotential was “negative”. The aPt/RuO_2NR_/Carbon exhibited 260-fold higher Pt mass activity (*i*_mPt_)/turnover frequency (TOF) (522.7 A mg^–1^/528.4 s^–1^) than that of commercial Pt/C (1.9 A mg^–1^/1.9 s^–1^). In an ultralow Pt loading (0.19 μg cm^–2^), the HER rate-determining step maintained Volmer–Tafel and the Pt utilization efficiency was 100.3%.

## Introduction

Hydrogen gas generated through electrochemical water splitting is a promising clean energy carrier that can replace fossil fuels. By converting renewable energy sources such as solar, wind, and hydropower into electricity, water electrolysis can produce high-purity hydrogen and oxygen. This method exhibits several advantages, including the use of plentiful resources, straightforward processes, no carbon emissions, and eco-friendliness, leading to its designation as “green hydrogen” production. Given these attributes, it is projected that “green hydrogen” will replace approximately 30% of “gray hydrogen” by 2030.^1−9^ Developing affordable and effective electrocatalysts for water splitting is crucial for a hydrogen economy. Although Pt is a highly efficient catalyst for the hydrogen evolution reaction (HER), its scarcity and cost hinder its widespread use. Therefore, reducing the Pt content of catalysts while maintaining a high catalytic activity is crucial. Traditional Pt-based catalysts (Pt-catalysts) often exhibit low utilization efficiency for Pt atoms (aPts). Single-atom catalysts (SACs), wherein individual metal atoms are dispersed and anchored onto a support, have the potential to achieve metal utilization rates of up to 100%. aPts-based SACs (Pt-SACs) provide a solution by minimizing the Pt loading and maximizing Pt activities, including the Pt mass activity (*i*_mPt_) and turnover frequency (TOF).^2,7,10−21^

The Volmer–Tafel mechanism (V–T) is the dominant HER rate-determining step (RDS_H_) for Pt catalysts in acidic solutions.^22,23^ In V–T, two active H atoms (H*) on the Pt surface form a H_2_ molecule via chemical bonding without electron transfer; this reduces the limitation of electron transfer kinetics on the HER (Scheme S1). The Tafel slope of the Tafel plot, typically set at 30 mV dec^–1^ at 25 °C, represents the standard value for V–T when evaluating the kinetics of the HER.^24^ The distance between adjacent H*-adsorbed Pt active sites (*D*_ee_) influences the bonding of the two H* atoms (Scheme S1).^25−30^ The RDS_H_ of the micrometer-sized XC-72 graphite carbon (C)-supported Pt (∼5 nm) catalysts (Pt_*m*_/C; *m* represents the Pt wt %, with values of 10, 20, and 40%) shifts from V–T to the Volmer–Heyrovsky mechanism (V–H) as the Pt loading on the electrode decreases (Data S1, Figures S1 and S2). V–H occurs when H* continually reacts with H^+^ and electrons to form H_2_ molecules.^24^ The apparent activation energy of V–H is generally higher than that of V–T. Further reduction of Pt loading in HERs is limited in Pt_*m*_/C (Data S1 shown in the Supporting Information). Analysis of the underpotentially deposited H (H_upd_) region using cyclic voltammetry generally shows characteristic H_upd_ signals for Pt catalysts, which are indicative of the generation of adsorbed H (H_ad_) (Figure S3).^23,24^ No H_upd_ signals are observed for most Pt-SACs. The absence of H_upd_ signals is attributed to the atomic-sized Pt.^31^ The aggregation of aPts diminishes catalytic activity. Generally, this is avoided by reducing aPt density and stabilizing the aPts on the support surface when designing Pt-SACs.^32^ However, the sparse distribution of aPts in Pt-SACs lengthening *D*_ee_ results in the V–H pathway of RDS_H_ (Tafel slopes > 30 mV dec^–1^).

This study proposed RuO_2_ nanorods (RuO_2NR_s) with lateral dimensions of 2–5 nm and rod lengths of 50–150 nm as nanometer-sized alternatives to micrometer-sized supports.^33^ Nanometer-sized spatial confinement shortens the *D*_ee_. The O atoms on RuO_2NR_s, similar to common metal oxides, stabilize the aPts. However, they increase the risk of aPt aggregation compared with micrometer-sized spatial confinement. This concept was actualized using the carbon-promoted spontaneous generation of aPts on RuO_2_. To the best of our knowledge, this was the first observation of the spontaneous and selective formation of aPts on RuO_2_; however, this was not observed on carbon without the addition of reductants in a mixture of C and RuO_2_ powder (C + RuO_2_). RuO_2_ acts as a charge mediator and a support for the aPts. The size of the aPts was limited to the atomic size owing to the controllable Pt growth rate during spontaneous generation. The process was extended to carbon-supported RuO_2NR_ (RuO_2NR_/Carbon), which comprised C, multiwall carbon nanotube (MWCNT), and graphene (GE).^33^ High-density aPts (HD-aPts) were embedded in RuO_2NR_ in RuO_2NR_/Carbon (aPt/RuO_2NR_/C, aPt/RuO_2NR_/MWCNT, and aPt/RuO_2NR_/GE). The HER onset potential (*E*_i_) on aPt/RuO_2NR_/Carbon was more positive than the standard hydrogen reduction potential (E^0^(H^+^/H_2_) = 0.0 V); therefore, the HER onset overpotential (η_i_ = *E*^0^(H^+^/H_2_) – *E*_i_) was “negative”. Similar to most Pt-SACs, H_upd_ signals were also absent. However, H_upd_ occurs on aPt/RuO_2NR_/Carbon owing to HD-aPts, which results in a negative η_i_ in HER. This is the first instance of Pt-SACs retaining H_upd_. The RDS_H_ on aPt/RuO_2NR_/Carbon exhibited V–T even under conditions of ultralow Pt loadings (<0.19 μg cm^–2^). aPt/RuO_2NR_/Carbon exhibited a 260-fold higher *i*_mPt_/TOF (522.7 A mg^–1^/528.4 s^–1^) than Pt_20_/C (1.9 A mg^–1^/1.9 s^–1^) at an overpotential, η, of 60 mV (η = *E*^0^(H^+^/H_2_) – *E*_,_ where *E* is the applied potential vs the reversible H electrode (RHE)). H_upd_ and CO-specific adsorption/desorption charge densities, determined using cyclic voltammetry, are typically used to assess the Pt-specific surface area (SA_Pt_ = Pt surface area/Pt mass).^24,34^ However, discussions surrounding H_upd_ and CO-specific adsorption/desorption for SA_Pt_ assessments (SA_HPt_ and SA_COPt_) were limited for most Pt-SACs.^34,35^ Surprisingly, the SA_HPt_ and SA_COPt_ on aPt/RuO_2NR_/Carbon were 1190.5 m^2^/g and 1085.1 m^2^/g, respectively, which are superior to those of common Pt catalysts (30–120 m^2^/g) and similar to the theoretical SA_Pt_ of aPt (SA_t-aPt_ = 1187.1 m^2^/g, the Pt atomic surface area calculated using the Pt van der Waals radius of 175 pm per Pt atomic mass). The SA_HPt_ to SA_t-aPt_ ratio showed that the Pt utilization efficiency (U_HPt_) of aPt/RuO_2NR_/Carbon reached 100.3%.

## Experimental Section

### Chemicals

MWCNT (purity >95%, outer diameter: 20–40 nm and length: 5–15 μm, specific surface area = 40–300 m^2^/g), GE (particle size: 8 nm), Pt on XC-72 Vulcan carbon (Pt_*m*_/C with 10, 20, and 40 wt % Pt, E-TEK), and graphite powder (XC-72) were purchased from Unionward Corp. Tannic acid (TA), ruthenium(III) chloride hydrate (RuCl_3_·*x*H_2_O), 99.9% ruthenium oxide (RuO_2_), 5% Nafion perfluorinated resin in a mixture of lower aliphatic alcohols and water (5% NF), and 95–98% H_2_SO_4_ (Aldrich) were purchased from Sigma-Aldrich. All chemicals were of analytical grade and used as received without further purification.

### Preparation of C(Pt^2+^), RuO_2_(Pt^2+^), and C + RuO_2_(Pt^2+^)

C and RuO_2_ powders were weighed separately to maintain a Ru content of 5%. Both samples were placed in an agate mortar and ground uniformly to obtain a mixture of C and RuO_2_ powder (C + RuO_2_). Catalysts (20 mg; C, RuO_2_, and C + RuO_2_ (with 5 wt % Ru content)) were separately incubated in 15 mL of 0.5 mM Pt^2+^ aqueous solution (Pt_aq_) at room temperature (∼28 °C) for 8 h. Following centrifugation, the samples were washed with deionized water and centrifuged. The as-prepared catalyst powders were dried under vacuum at 65 °C to obtain C(Pt^2+^), RuO_2_(Pt^2+^), and C + RuO_2_(Pt^2+^).

### Preparation of aPt/RuO_2NR_/Carbon

First, RuO_2NR_/Carbons were prepared using a previously published procedure.^33^ Carbon (20 mg), which included MWCNT, GE, and C, was dispersed into 15 mL of 0.78 mM TA aqueous solution and sonicated (50 W, 20 kHz) to obtain a TA multilayer-modified carbon suspension (0.12 wt %). Subsequently, 7 mL of 0.61 mM RuCl_3_ aqueous solution was added to 3 mL of TA_m_/C_aq_ and stirred for 30 min. The solution pH was gradually tuned to 10 using 1.67 mM NaOH to obtain a TA monolayer-modified carbon (TA_i_/Carbon). The TA_i_/Carbon accumulated the Ru(OH)_2_^+^ precursor (Ru(OH)_2ads_/TA_i_/Carbon) under alkaline conditions. The as-prepared Ru(OH)_2ads_/TA_i_/Carbon was dried under vacuum at 65 °C for 12 h followed by thermal treatment in an Ar flow (0.5 cc min^–1^) at 250 °C for 2 h to obtain RuO_2NR_/Carbon. The as-prepared RuO_2NR_/Carbon (15 mg) was incubated in 15 mL of 0.5 mM Pt_aq_ at ∼28 °C for 8 h. Following centrifugation, the samples were washed with deionized water and centrifuged. As-prepared catalyst powders were then dried under vacuum at 65 °C; aPt/RuO_2NB_/Carbons (aPt/RuO_2NB_/C, aPt/RuO_2NB_/MWCNT, and aPt/RuO_2NB_/GE) were obtained.

### Characterization

Surface elements on catalysts were analyzed by X-ray photoelectron spectroscopy (XPS). The XPS data were acquired using a ULVAC-PHI, PHI5000 VersaProbe/scanning ESCA microprobe. In X-ray diffraction (XRD) measurements (Cu K_α_ radiation, λ = 1.54 Å), a Ti foil (10 × 10 mm) was used as an electrocatalyst holder. Inductively coupled plasma mass spectrometry (ICP-MS; Agilent 7500ce instrument) was used to determine the metal loading on the catalysts. The samples were incubated overnight in 2 mL of aqua regia in preparation for ICP-MS analysis. The aqua regia was diluted using deionized water and then boiled to remove HCl from the solution. After readding water and boiling it several times, the remaining solution was diluted to a suitable concentration for ICP-MS analysis. Thermogravimetric analysis (TGA) was used to confirm the Ru and Pt content of aPt/RuO_2NB_/Carbons and was conducted using TGA/DSC equipment (Mettler-Toledo, 2-HT) at a nitrogen or air purge rate of 30 mL/min to assess the thermal stability of the catalysts. Micromorphological analysis of the catalysts was performed using a JEOL JEM-1400 transmission electron microscope and a JEOL JEM-2100F field-emission transmission electron microscope. High-resolution transmission electron microscopy (HRTEM) images were obtained using FE-TEM. The Pt L_3_-edge and Ru K-edge X-ray absorption near-edge structure (XANES) and extended X-ray fine structure (EXAFS) analyses were performed using the TPS 44A beamline at the National Synchrotron Radiation Research Center, Taiwan.^36^ Data processing was performed according to standard procedures using the Demeter software package.

### Electrochemical Experiments

Electrochemical experiments were performed using a CHI 760C potentiostat/galvanostat and three-electrode electrochemical cell. Hg/HgSO_4_ was used as the reference electrode in acidic aqueous solutions to prevent interference due to Cl^–^ from the Ag/AgCl reference electrode. A graphite rod was used as the counter electrode to prevent the interference of Pt ions from the common Pt counter electrode. The potentials measured against the Hg/HgSO_4_ reference electrode were converted to a reversible H electrode (RHE) reference scale using *E*_RHE_ = *E*_Hg/HgSO4_ + 0.68 + 0.059pH_electrolyte_. All potentials in this paper are stated with reference to the RHE. A glassy carbon electrode (GCE, 0.07 cm^2^ for examination of the electrocatalyst performance) served as the substrate electrode for the electrocatalyst suspension. The electrocatalyst suspensions were prepared by weighing 2 mg of electrocatalyst followed by the addition of 0.83 mL of deionized water (with specific resistivity of 18.2 MΩ cm). Subsequently, the gradual addition of 0.17 mL of isopropyl alcohol, 5 μL of a 5% NF, and 20 min of ultrasonication resulted in the formation of an electrocatalyst suspension ink. The CVs were recorded using a catalyst thin film-covered GC disk electrode (catalyst/GCE) in an Ar-purged 0.5 M H_2_SO_4_ aqueous solution (H_2_SO_4aq_). The catalyst/GCE was fabricated using the drop-coating procedure. Briefly, a GCE was polished successively with 1.0, 0.3, and 0.05 μm alumina powder cloths (Buchler) followed by sonication in deionized water and drying. A 4 μL solution of the electrocatalyst suspension ink was pipetted onto the surface of GCE (Pt loading is 0.17–0.43 μg cm^–2^). The catalyst/GCE was dried under Ar flow at room temperature to evaporate the solvent. Thereafter, 5.0 mL of 0.5 M H_2_SO_4aq_ was used as the aqueous electrolyte solution for the electrochemical measurements. This solution was degassed by bubbling Ar for 5 min before analysis. The charge densities from the H_upd_ and CO-specific adsorption/desorption in CVs (Q_H-upd_ and Q_CO-stripping_) were used to assess the Pt surface area (Figures S21 and S22). A conversion factor of 0.21 mC cm^–2^ was used to determine the Pt surface area based on Q_H-upd_. For CO stripping experiments, CO was adsorbed on to the precleaned electrode by holding the potential at 0.05 V for 10 min in CO-saturated 0.1 M HClO_4_ solution. The CO stripping curve was taken after purging with Ar for 30 min. A conversion factor of 0.42 mC cm^–2^ was used to determine the Pt surface area based on Q_CO-stripping_. By plotting overpotential η against log (*j*) from linear scan voltammetry (LSV) curves, Tafel slopes were obtained. EIS measurements were performed in Ar-purged 0.5 M H_2_SO_4aq_ η = 20 mV for the HER in the frequency range of 100 kHz to 0.1 Hz.

The turnover frequency (TOF) was calculated according to the following equation:
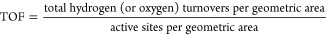

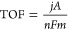
where *j* is the current density at a given overpotential (50, 60, or 70 mV), *A* is the surface area of the electrode, *n* = 2 is the number of electrons required for the HER, *F* is the Faraday constant, and *m* is the number of moles of Pt on the electrode.

For, the stability of aPt/RuO_2NR_/Carbon in the HER process, the detailed results are depicted in Data S3 in the Supporting Information.

## Results and Discussion

**Figure 1:**
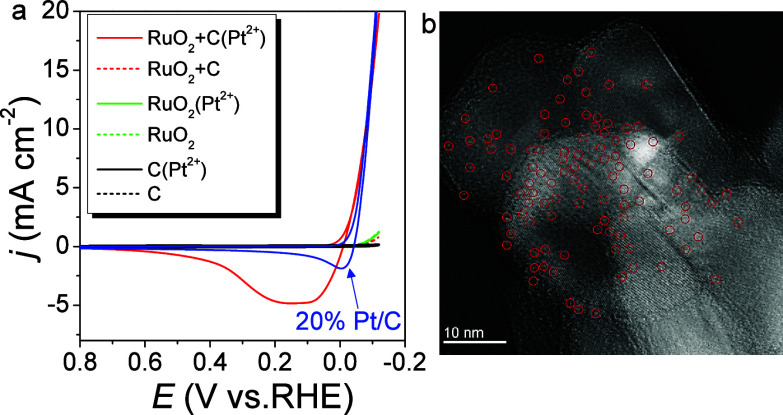
(a) CVs of C, RuO_2_, and C + RuO_2_ (Ru content is 5 wt %) before (dashed line) of C and RuO_2_ after incubation with Pt_aq_ (C(Pt^2+^) and RuO_2_(Pt^2+^)). C + RuO_2_ after incubation in Pt_aq_ (C + RuO_2_(Pt^2+^)) exhibits a sharp reduction current starting at *E*_i_ = 0.024 V vs RHE. This is accompanied by the generation of a large quantity of H_2_ bubbles. and after (solid line) incubation in Pt_aq_ for 8 h in Ar-saturated 0.5 M H_2_SO_4aq_ at a scan rate of 50 mVs^–1^. (blue line) A CV of 20% Pt/C was used as a reference result. (b) HAADF-STEM image of C + RuO_2_(Pt^+^) (aPts are highlighted by red circles).

Cyclic voltammograms (CVs) of catalysts, including C, RuO_2_, and C + RuO_2_ (Ru content is 5 wt %), before and after incubation in 0.5 mM Pt^2+^ aqueous solution (Pt_aq_) at room temperature (∼28 °C) for 8 h are shown in Figure 1. The CVs were recorded using a catalyst thin film-covered glassy carbon disk electrode (catalyst/GCE) in an Ar-purged 0.5 M H_2_SO_4_ aqueous solution (H_2_SO_4aq_). No changes are observed in the CVs.

The HER on the C + RuO_2_(Pt^2+^) occurs at a more positive *E*_i_ than that on the Pt_20_/C (−0.015 V) and *E*^0^(H^+^/H_2_). The HER η_i_ on the C + RuO_2_(Pt^2+^) is “negative”. Bright-field high-resolution transmission electron microscopy (HR-TEM) images and the corresponding high-angle annular dark-field scanning TEM (HAADF-STEM) images of C + RuO_2_(Pt^2+^) indicate that the RuO_2_ surface is covered with aPts (Figure S4 and Figure 1b). The average size of the aPts is close to the atomic size (average diameter, *D*_av_, ∼0.4 nm). These aPts are observed as ultrasmall dark and bright dots in HR-TEM and HAADF-STEM images, respectively. The aPts are solely detected on RuO_2_ in the energy-dispersive spectroscopy (EDS) elemental mapping of the TEM image of C + RuO_2_(Pt^2+^) (Figure S5). Inductively coupled plasma mass spectrometry (ICP-MS) shows that the Pt wt % in C + RuO_2_(Pt^2+^) is ∼0.15 wt %. These results indicate that aPts form spontaneously and selectively on RuO_2_ in C + RuO_2_(Pt^2+^) in the absence of additional reductants. Additionally, as shown in Figure 1, within the same reduction potential range (−0.15 to 0.0 V), RuO_2_ and C do not show a clear HER activity contribution. It is only after the induction of aPt that a significant HER performance begins to appear, which supports the decisive contribution of aPt to HER activity in this operating potential range.

The elemental states of C, C + RuO_2_, and C + RuO_2_(Pt^2+^) were investigated using X-ray photoelectron spectroscopy (XPS) (Figure S6). The peaks of C 1s for C at binding energies (BEs) of ∼283.2/284.0 eV shifted to higher BEs (283.6/284.2 eV) in C + RuO_2_ and then returned to ∼283.2/284.0 eV in C + RuO_2_(Pt^2+^) (Figure S6a). The O 1s peak intensity ratio (C=O/C–O) in C + RuO_2_ (1.9) was higher than that in C (1.04); subsequently, it decreased to 1.08 in C + RuO_2_(Pt^2+^) (Figure S6b). RuO_2_ increased the oxidation state of C in C + RuO_2_; however, C returned to its original oxidation state in C + RuO_2_(Pt^2+^). Two Pt 4f_7/2_/4f_5/2_ peaks were observed at higher BEs (∼71.4/74.8 eV) in C + RuO_2_(Pt^2+^) than those in Pt_20_/C (∼70.6/74.0 eV) (Figure S6d). The shifts in the Pt 4f_7/2_/4f_5/2_ peaks indicated a partial oxidation of aPt within C + RuO_2_(Pt^2+^). The O 1s peak intensity of Ru–O (∼529.6 eV) decreased in C + RuO_2_(Pt^2+^) (Figure S6b). The aPts formed on RuO_2_ in C + RuO_2_(Pt^2+^) generated a Pt–O interaction and then weakened the Ru–O interaction. The unchanged Ru 3p_3/2_/3p_1/2_ (∼463.2/485.6 eV) indicated that RuO_2_ only facilitated the transfer of electrons from C to Pt^2+^ (Figure S6c).

**Figure 2:**
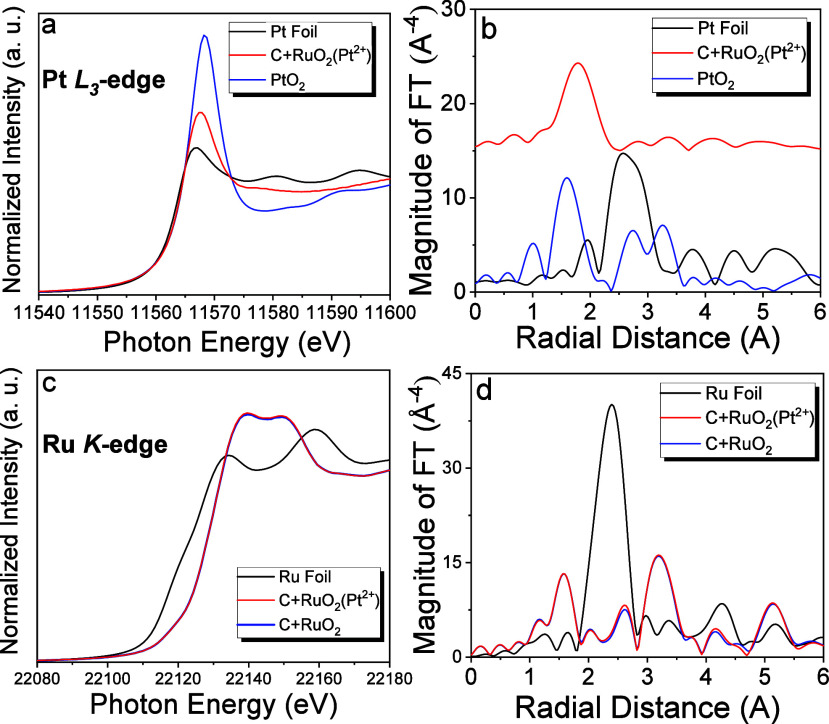
(a) Pt L3-edge and (c) Ru K-edge XANES spectra and (b, d) FT-EXAFS oscillations of C + RuO_2_, C + RuO_2_(Pt^2+^), Pt foil, PtO_2_, and Ru foil.

Examination of the Pt L3-edge and Ru K-edge using X-ray absorption near-edge structure (XANES) and extended X-ray fine structure (EXAFS) spectra revealed the electronic structure and coordination environments of the Pt and Ru atoms in C + RuO_2_(Pt^2+^) at the atomic level (Figure 2). Normalized Pt L_3_-edge XANES spectra show that the white-line intensity of C + RuO_2_(Pt^2+^) is between those of PtO_2_ and the Pt foil (Figure 2a), suggesting that aPts carry partially positive charges.^36^Figure 2b shows the Pt L_3_-edge Fourier transforms of the EXAFS (FT-EXAFS) oscillations of the related samples (*k*^3^-weighted χ(*k*) signals in Figure S7a), in which the Pt–Pt contribution at approximately 2.5 Å for Pt foil is absent in C + RuO_2_(Pt^2+^). This indicates that no Pt nanoparticles or clusters are present in C + RuO_2_(Pt^2+^). Alternatively, the only prominent shell, located at 1.78 Å, corresponds to a Pt–O bond, which is longer than that in PtO_2_ (1.6 Å). Based on the above analysis, the aPt is stabilized on RuO_2_ by Pt–O interactions with the surrounding O. Moreover, the Ru K-edge XANES and EXAFS of C + RuO_2_ are unaffected in C + RuO_2_(Pt^2+^), indicating that RuO_2_ only acts as a charge mediator in the aPt formation process (detailed fitting results and fitting parameters are shown in Figure S30 and Table S1).

**Figure 3:**
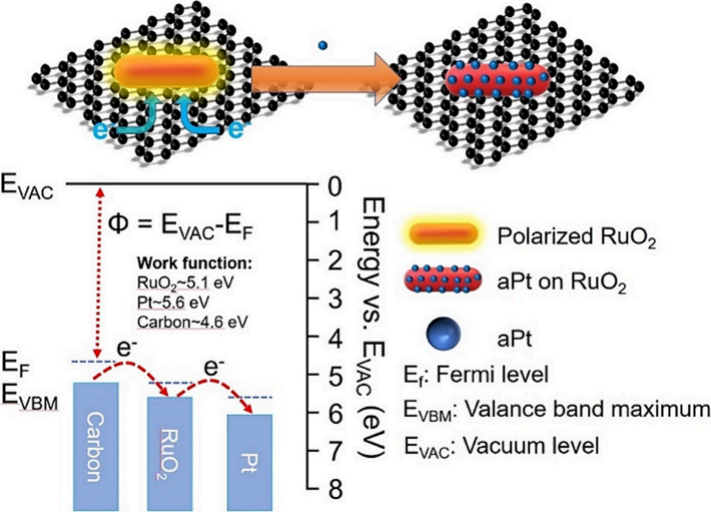
Schematic model for the carbon-promoted aPt spontaneous generation on RuO_2_.

**Figure 4:**
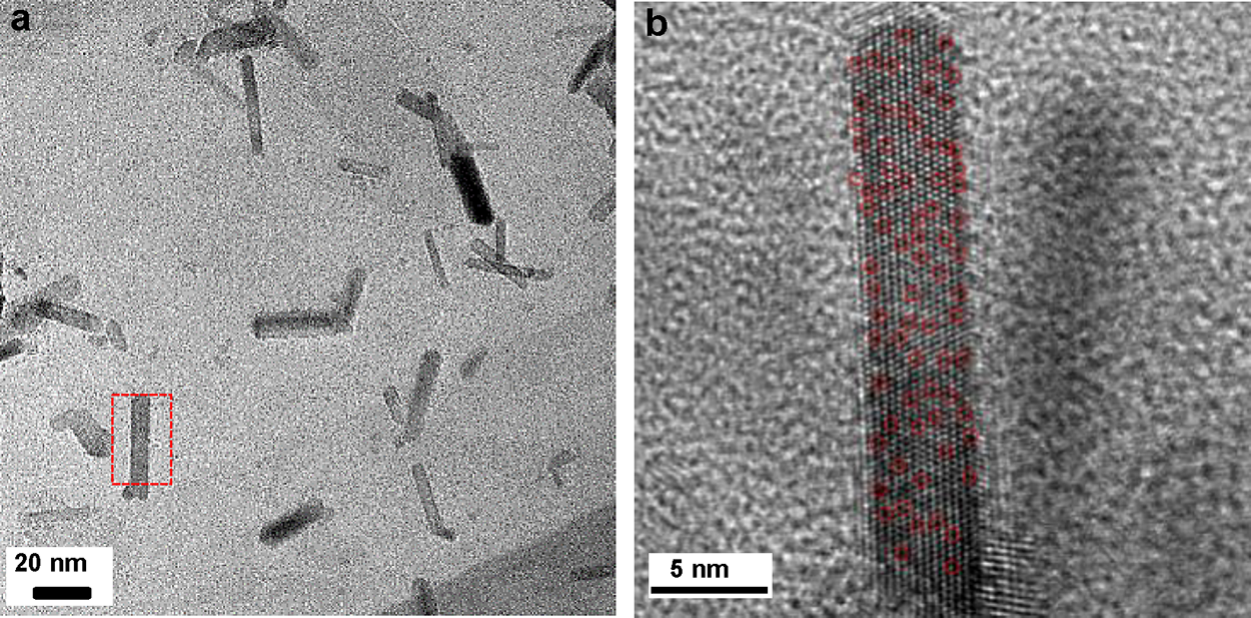
(a) TEM image of aPt/RuO_2NR_/GE. (b) HR-TEM image of one aPt/RuO_2NR_ in the marked area in (a) (aPts are selectively marked by red circles).

To explain this process, a model was proposed (Figure 3). RuO_2_ polarizes the C surface, and electrons migrate toward the valence band maximum (*E*_VBM_) of RuO_2_ in C + RuO_2_. This is because the work function (Φ) of RuO_2_ (∼5.1 eV) is higher than that of C (∼4.6 eV).^37,38^ Electron migration does not affect the electronic configuration of Ru but increases the oxidation state of C. The increased electron density of RuO_2_ reduces aPts on RuO_2_. Once aPts are formed, electron migration from C to RuO_2_ ends, thereby limiting the growth of aPts and controlling their sizes. Additionally, the reduction of Pt to C is a kinetically limited reaction owing to its high activation energy barrier. Therefore, RuO_2_ acts as an electron mediator, directing electrons from C and promoting aPt formation on RuO_2_. RuO_2NR_/Carbon, comprising C, MWCNT, and GE, was fabricated in a previous study (Figures S8 and S9).^33^ Extending the proposed model RuO_2NR_/Carbon was incubated in Pt_aq_ to obtain a series of aPt/RuO_2NR_/Carbon (Figure 4 and Figures S10 and S11). Although the Pt wt % in RuO_2NR_/Carbon was ultralow (∼0.16 wt %), aPts (*D*_av_ = ∼0.36 nm) were full of each RuO_2NR_ (EDS mapping shown in Figure S12). By tracking the RuO_2_ and Pt content variation in RuO_2NR_/Carbon throughout the incubation time, variations in Pt wt % were observed. The Pt wt % was lower than ∼0.12 wt % before 8 h and then plateaued at ∼0.16 wt %. Additionally, the RuO_2_ content was largely unaffected (Figure S13), suggesting that the formation of aPts was guided by the RuO_2NR_ mediating electrons from carbon.

**Figure 5:**
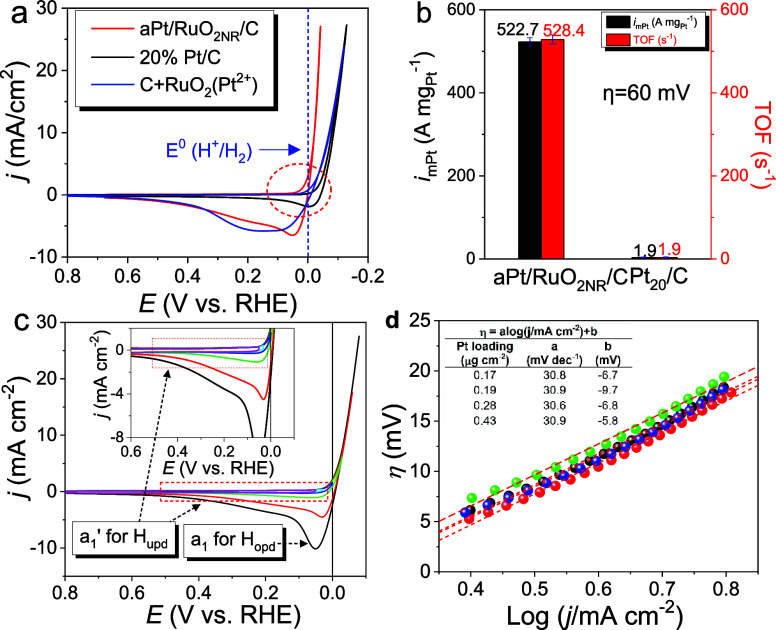
(a) CVs of aPt/RuO_2NR_/C, Pt/C, and C + RuO_2_(Pt^2+^) in Ar-saturated 0.5 M H_2_SO_4aq_ at a scan rate of 50 mVs^–1^. (b) *i*_mPt_/TOF for aPt/RuO_2NR_/C and Pt/C at a η of 60 mV. (c) CVs of aPt/RuO_2NR_/C recorded at various reversed potentials in Ar-purged 0.5 M H_2_SO_4_. (d) Tafel plots for aPt/RuO_2NB_/C recorded in various Pt loadings.

The CVs of aPt/RuO_2NR_/Carbon/GCE exhibited excellent HER performances, which were superior to that of Pt_20_/C (Figure S14). The HER activity on aPt/RuO_2NR_/Carbon was greater than that on C + RuO_2_(Pt^2+^) because of the more homogeneous distribution and stronger interaction of RuO_2NR_ with carbon (Figure 5a). Charge-transfer resistance (*R*_ct_) is related to the electrocatalytic kinetics of HER. The Nyquist plots of the electrochemical impedance spectra (EIS) (Figure S15) showed that the *R*_ct_ of aPt/RuO_2NR_/Carbon (3.8 Ω cm^2^) was lower than those of RuO_2NR_/C (142.2 Ω cm^2^) and Pt_20_/C (46.6 Ω cm^2^). This highlighted the superior charge transfer of aPt/RuO_2NR_/Carbon during HER in acidic solutions. The Pt and Ru XPS signals of aPt/RuO_2NR_/Carbon exhibited the same trend as those of C + RuO_2_(Pt^2+^) (Figure S16). C 1s and O 1s shifted to higher BEs in RuO_2NR_/Carbon than that in C + RuO_2_, indicating stronger RuO_2NR_–carbon interactions. The Pt–O peak of the O 1s spectrum indicated that HD-aPts were formed on the RuO_2NR_ surface (Figure S16b). Pt L_3_-edge, Ru K-edge XANES, and EXAFS of aPt/RuO_2NR_/Carbon also suggested that HD-aPts were stabilized on RuO_2NR_ (Figures S17 and S18). The Pt^2+^ concentration and incubation time weakly affected the amount and size of aPt particles on RuO_2NR_ after 16 h. Additionally, the RuO_2_ content was largely unaffected (Figure S13), suggesting that the formation of aPts was guided by the RuO_2NR_ mediating electrons from carbon.

Three parameters were evaluated for the HER activity (Figure 5b); TOF, *i*_mPt_ (specific currents at η = 60 mV (50 mV and 70 mV shown in Figure S19) on the HER polarization curves normalized by the moles and the mass of Pt), and η_10_ (η at a specific current density of 10 mA cm^–2^) are listed in Table S2. aPt/RuO_2NR_/C exhibited outstanding HER performances, including an ultralow η_10_ (18 mV) that was 29 mV lower than that of Pt_20_/C (47.1 mV); additionally, a significant increase in *i*_mPt_/TOF (522.7 A mg^–1^/528.4 s^–1^), which was 280-fold higher than that of Pt_20_/C (1.9 A mg^–1^/1.9 s^–1^), was observed. These high values of *i*_mPt_ and TOF were superior to that of the modern Pt-SACs (0.204–26.1 A mg^–1^ and 0.1–41 s^–1^, shown in Figure S20 and Table S2). While the prices of precious metals fluctuate rapidly, the price difference between Pt and Ru, which is approximately 2–4 times, should be a fact. We have based our price activity estimation on the prices of Ru ($14,146/kg) and Pt ($28,932/kg) as found on a Web site dated 3/21/2024. We also included the original Pt mass activity data (Figure 5b) into the RuO_2_ (5 wt %) for estimating the catalyst’s cost contribution. The related estimated price activities have been shown in the Supporting Information (Figure S29). The calculation of price activity can refer to the work of Guan et al.^39^

Similar to most Pt-SACs, the absence of H_upd_ signals in the CVs of aPt/RuO_2NR_/Carbon suggested that aPts were of atomic size (Figure 5a). In addition to the ultrahigh HER performances, aPt/RuO_2NR_/Carbon exhibited a “negative η_i_” during HER. Generally, H_upd_ was not observed on Pt-SACs; additionally, a “positive η_i_” was required for the HER to occur on its surface. CVs of aPt/RuO_2NR_/Carbon were recorded at various reversed potentials in Ar-purged 0.5 M H_2_SO_4_ (Figure 5c). By tracking the hydrogen oxidation reaction (HOR) signals in CVs, an oxidative peak, a_1_, with a shoulder wave, a_1_’, was observed in the anodic reverse potential sweeping after the cathodic generation of H_2_. When the reverse potential was above 0.0 V, only a_1_’ was observed; however, when it was below 0.0 V, a_1_’ and a_1_ were observed simultaneously. This indicated that a_1_’ was related to H_upd_ oxidation, whereas a_1_ originated from the overpotential deposition of hydrogen (H_opd_). Anodic linear scan voltammetry was used to examine the anodic wave, a_1_’, from H_upd_ oxidation. The linear scan voltammograms (LSVs) were recorded based on how long the potential was held at 0.04 V vs RHE (Figure S21). The anodic charge on a_1_’ saturated after 15 s; no a_1_ was observed. The limited anodic charge supported a_1_’, which corresponds to the anodic stripping of H_upd_. The anodic charge on a_1_’ depends on the surface area of the aPts. Charge densities related to the adsorption/desorption of H_upd_ and CO in CVs were used to estimate SA_HPt_ and SA_COPt_. The SA_HPt_ and SA_COPt_ on aPt/RuO_2NR_/Carbon were measured as 1190.5 m^2^/g and 1085.1 m^2^/g (Figures S22 and S23), respectively, which were superior to those of common Pt catalysts (30–120 m^2^/g) and close to SA_t-aPt_ (1187.1 m^2^/g). Based on the SA_HPt_ to SA_t-aPt_ ratio, the Pt utilization efficiency (U_HPt_) of aPt/RuO_2NR_/C reached 100.3%. HER polarization curves and associated Tafel plots were recorded to monitor the Pt loading effect (0.17–0.43 μg cm^–2^) on the HER performance of aPt/RuO_2NR_/CarbonC (Figure S24 and Figure 5d). Because aPts are only formed on RuO_2NR_, the Pt contents (0.15–0.38%) were adjusted by varying the RuO_2NR_ content on aPt/RuO_2NR_/Carbon (Figure S25). With reduced Pt loading, the HER performance remained consistent, and the corresponding Tafel plots continued exhibiting Tafel slopes of 30 mV dec^–1^. The RDS_H_ on aPt/RuO_2NR_/Carbon remained V–T even at ultralow Pt loadings (<0.19 μg cm^–2^). This was attributed to the spatial constraint of HD-aPts on RuO_2NR_, which reduced *D*_ee_ and enhanced the “interparticle” or “inter-aPts” Tafel possibility. The V–T mechanism observed for aPt/RuO_2NR_/Carbon indicated that it was kinetically favored in the HER, even at ultralow Pt loadings

## Conclusions

The challenge associated with Pt catalysts and Pt-SACs lies in maintaining the kinetically favored V–T in the HER at low Pt loadings. A pivotal factor, *D*_ee_, was reduced owing to the spatial confinement within the nanometer-sized support, RuO_2NR_. This confinement augments the potential of “inter-aPts” Tafel reactions. aPt/RuO_2NR_/Carbon delivers a high HER performance and pioneers the retention of H_upd_ on Pt-SACs. Even at minimal Pt loadings, RDS_H_ on aPt/RuO_2NR_/Carbon exhibited V–T. The SA_HPt_ and SA_COPt_ values stand at 1190.5 m^2^/g and 1085.1 m^2^/g, respectively; these values were similar to SA_t-aPt_. Additionally, a U_HPt_ value of 100.3% was achieved on aPt/RuO_2NR_/Carbon. The discovery of spontaneous carbon-promoted aPt generation on RuO_2_ has paved the way for the formation of HD-aPts on metal oxides. This innovative process offers potential avenues for the development of other metal oxide-supported aPt catalysts, expanding their utility in diverse applications.

## Supporting Information Available

Data S1–S3: influences of Pt loading for the HER mechanism on Pt_*m*_/C, electrochemical determination of *D*_ee_, and stability of aPt/RuO_2NR_/Carbon in HER process; Scheme S1: *D*_ee_ influences the bond formation between two H* and the HER mechanism. Figures S1–S30: Tafel plots of Pt_*m*_/C, HR-TEM images of Pt_*m*_/C, CVs of the Pt_*m*_/C/GCE, EDS mapping results, high-resolution XPS spectra of C + RuO_2_(Pt^2+^), C + RuO_2_, C, and Pt/C, *k*^3^-weighted FT-EXAFS spectra, RuO_2_ and Pt content in aPt/RuO_2NR_/Carbon, EIS results, *i*_mPt_/TOF for aPt/RuO_2NR_/C, *i*_mPt_ for the modern Pt-SACs, anodic LSVs for H_upd_ on aPt/RuO_2NR_/C, CO stripping curves on aPt/RuO_2_/C, tracking RuO_2_ content by CVs of aPt/RuO_2NR_/C, stability tests of aPt/RuO_2NR_/Carbon in HER through CV scanning and XRD analysis, and price activities of aPt/RuO_2NR_/C; Tables S1 and S2: best-fit FT-EXAFS parameters of aPt/RuO_2_/C catalysts and HER performances of Pt-SACs and Pt catalysts (PDF)
