# Supplementary material for: Carbon-Promoted Pt-Single Atoms Anchored on RuO2 Nanorods to Boost Electrochemical Hydrogen Evolution
Source: ACS Appl Mater Interfaces. 2024 May 17;16(21):27504–10. doi: 10.1021/acsami.4c06033 (PMC11145582; doi:10.1021/acsami.4c06033)
Supplement: Supplementary file 1 — am4c06033_si_001.pdf [file am4c06033_si_001.pdf]

Supporting Information

Carbon-Promoted Pt-Single Atoms Anchored on RuO_2 Nanorods to Boost Electrochemical Hydrogen Evolution

Jing-Fang Huang,*[a] Wen-Jun Hsieh,[a] Jeng-Lung Chen[b]

[a] Department of Chemistry, National Chung Hsing University, Taichung 402, Taiwan
[b] National Synchrotron Radiation Research Center, Science-Based Industrial Park, Hsinchu 30076, Taiwan
Correspondence to: jfh@dragon.nchu.edu.tw

This PDF file includes:

Data S1-S3:

Influences of Pt loading for HER mechanism on Pt_m/C, electrochemical determination of D_{ee}, and the stability of aPt/RuO_{2NR}/Carbon in HER process.

Scheme S1:

D_{ee} influences the bond formation between two H* and the HER mechanism.

Figures. S1-S30:

Tafel plots of Pt_m/C, HR-TEM images of Pt_m/C, CVs of the Pt_m/C/GCE, EDS mapping results, high-resolution XPS spectra of C+RuO_2(Pt^{2+}), C+RuO_2, C and Pt/C, k^3-weighted FT-EXAFS spectra, RuO_2 and Pt content in aPt/RuO_{2NR}/Carbon, EIS results, i_{mPt}/TOF for aPt/RuO_{2NR}/C, i_{mPt} for the modern Pt-SACs, anodic LSVs for H_{upd} on aPt/RuO_{2NR}/C, CO stripping curves on aPt/RuO_2/C, tracking RuO_2 content by CVs of aPt/RuO_{2NR}/C, stability tests of aPt/RuO_{2NR}/Carbon in HER through the CV scanning and the XRD analysis, and price activities of aPt/RuO_{2NR}/C.

Tables S1 and S2:

The best-fit FT-EXAFS parameters of aPt/RuO_2/C catalysts and HER performances of Pt-SACs and Pt-catalysts.

Data S1

Influences of Pt loading for HER mechanism on Pt_m/C

The Volmer–Tafel mechanism (V-T) is the dominant HER rate-determining step (RDS_H) for Pt-catalysts in acidic aqueous solutions. During V-T, two active H atoms (H*) on the Pt surface form an H_2 molecule via chemical bonding without electron transfer. This results in the reduction of the HER by electron transfer kinetics (Scheme S1). The Tafel slope on the Tafel plot, typically set at 30 mV dec^{-1} at 25 °C, represents a standard value for V-T when evaluating the kinetics of HER. The HER polarization curves and corresponding Tafel plots of the micrometer-sized XC-72 graphite carbon-supported nanometer-sized Pt (~5 nm) catalysts (denoted as Pt_m/C, where m represents the Pt wt. %, with values of 10, 20, and 40) are shown in Figure S1. The η_{10} of the HER on Pt_m/C increases with decreasing Pt loading on the electrode. Based on the Tafel slope assessment, the RDS_H of Pt_m/C shifts from the V-T to the Volmer–Heyrovsky mechanism (V-H) as the Pt loading on the electrode decreases (Figures. S1 and S2). This transition is achieved by adjusting the catalyst ink dilution. V-H is H* that continually reacts with H^+ and electrons to form H_2 molecules. The distance between adjacent H*-adsorbed Pt active sites (D_{ee}) influences the bonding possibility of the two H* atoms (Scheme S1 and Figure S2). The HR-TEM images of Pt_m/C are shown in Figure S2. The average size of the Pt nanoparticles is ~5 nm. Assuming that the density of the Pt nanoparticles on each C particle is homogeneous, the final Pt loading on the electrode is varied by tuning the Pt_m/C ink concentration. The aggregation of Pt_m/C particles in high Pt loading (2~0.5 μg cm^{-2}) shortens the D_{ee}; however, the actual D_{ee} is observed at low Pt loading (<0.2 μg cm^{-2}) (Scheme S1 and Figure S2). D_{ee} is constrained by the spacing of Pt anchored to C in Pt_m/C. Pt_m/C is limited to further reducing the Pt loading in HER applications.

Data S2

Electrochemical determination of D_{ee}

According to our previous report, a combination of Pt-electrochemical surface area (ECSA) and the anodic charge (Q_{Pt}) from the anodic stripping of Pt during anodic CV scanning on Pt-based catalysts covering GCE was used to calculate the mean diameter of Pt particles (D_{Pt}) and the number of Pt particles on the substrate (N_{Pt}).[S1-S6] The method was also extended to calculate the Pt diameter in atomic size (less than 1 nm) in this study, as the retention of H_{upd} on a Pt/RuO_{2NR}/Carbon can be used to measure the electrochemical surface area (ECSA). The Pt content can be directly evaluated from Q_{Pt}, assuming four electrons are transferred per Pt atom. The Pt content evaluated directly from Q_{Pt} is consistent with the results obtained by ICP-MS and TGA. ECSA was determined by measuring the areas (charges) under the electro-oxidation of adsorbed H_{upd} (Figure S3). A conversion factor of 0.21 mC cm^{-2} was used to determine the ECSA. Assuming that the Pt particles are uniformly distributed and spherical in shape on the substrate, ECSA and Q_{Pt} can be calculated using equations (S1) and (S2), respectively.

$$\mathrm{ECSA} = \frac{\pi D_{Pt}^{2}}{2} \times N_{Pt} \quad \text{(S1)}$$

$$Q_{Pt} = \frac{\pi D_{Pt}^{3} \rho_{Pt}}{6 M_{Pt}} \times n \times F \times N_{Pt} \quad \text{(S2)}$$

where n = 4 represents the number of electrons transferred for the anodic stripping of Pt, F is the Faraday constant, ρ_{Pt} is the density of Pt (21.09 g cm^{-3}), and M_{Pt} is the atomic mass of Pt. The Q_{Pt}/ECSA ratio is used for evaluating the D_{Pt} of Pt as shown in equation (S3). N_{Pt} can then be obtained from D_{Pt}.

$$D_{Pt} = \frac{3 M_{Pt}}{4 F \rho_{Pt}} \times \frac{Q_{Pt}}{\mathrm{ECSA}_{CO}} \quad \text{(S3)}$$

The inter-particle distance (D_{ee}) represents the distance between two Pt particles (edge-to-edge distance between each Pt particle) on a substrate. We assumed that all Pt particles are spherical for the calculation of D_{ee}. The Pt particles were monodispersed and homogenously distributed on the carbon support (Scheme S1). Nitrogen adsorption and desorption studies were performed at 77 K on a Micromeritics TriStar 3000 adsorption apparatus. A_{BET} was measured using the BET method. The center-to-center distance between each particle (D_{cc}) was evaluated from $(A_{BET}/N_{Pt})^{0.5}$. D_{ee} is $(A_{BET}/N_{Pt})^{0.5}$ - D_{Pt}.

Data S3

The stability of aPt/RuO_{2NR}/Carbon in HER process

To assess its stability in the HER process, aPt/RuO_{2NR}/Carbon was subjected to continuous potential scanning, cycled 5000 times from 0.6 V to -0.1 V versus RHE, at a scan rate of 0.05 V/s. The polarization curves for the first and 5000th cycles show almost identical behaviors without any obvious change, as depicted in Figure S26. While Pt ions could transfer to the electrolyte due to the anodic potential being too positive (> 0.8 V versus RHE), they appear to remain stable during the cathodic operation in the HER process. ICP-MS was used to track the metal ions in the electrolyte solution. The content of Pt^{2+} and Ru^{3+} is less than 0.05 μM. This supports that the HER process did not cause the possible dissolution of Pt and RuO_2 during HER. The XRD of pristine aPt/RuO_{2NR}/C is added in Figure S27. The diffraction peaks of RuO_{2NR} were observed, but the diffraction signals of aPt were not detected, which supports the notion that the active sites of aPt are at the atomic scale. After the HER stability test, the diffraction peaks of RuO_{2NR} did not show significant changes, and there was still no appearance of aPt diffraction signals. This supports that the HER process did not cause the agglomeration of aPt to form Pt clusters.

For practical application, the stability of aPt/RuO_{2NR}/Carbon for HER was further evaluated at a large current density, ~120 mA cm^{-2} (the overpotential of 100 mV). Figure S28a shows that the chronoamperometric curve of aPt/RuO_{2NR}/Carbon only retained 50% after 1h. To track the stability of aPt/RuO_{2NR}/Carbon, CVs of aPt/RuO_{2NR}/Carbon after electrolysis static overpotentials of 100 mV for HER for 12h in 0.5 M H_2SO_{4aq} were provided in Figure S28b. Despite the current density dropping to 50% of its original value during the first hour of continuous HER operation, it remained stable for 12 hours, with no significant increase in Pt^{2+} and Ru^{3+} in the electrolyte solution. However, CV tracking results showed that the onset potential for HER shifted in the cathodic direction from 0.05 V to 0.0 V, which is close to the onset potential of bulk Pt, after the high current density HER test. Since there was no significant decrease in Pt content in the catalysts, the reduction in HER activity is likely due to the strong polarization of the electrode surface under high current density, leading to the migration and aggregation of Pt single atoms on the catalyst surface, forming larger Pt clusters. This issue needs to be addressed for the future application of aPt/RuO_{2NR}/Carbon in practical high-current density operations.

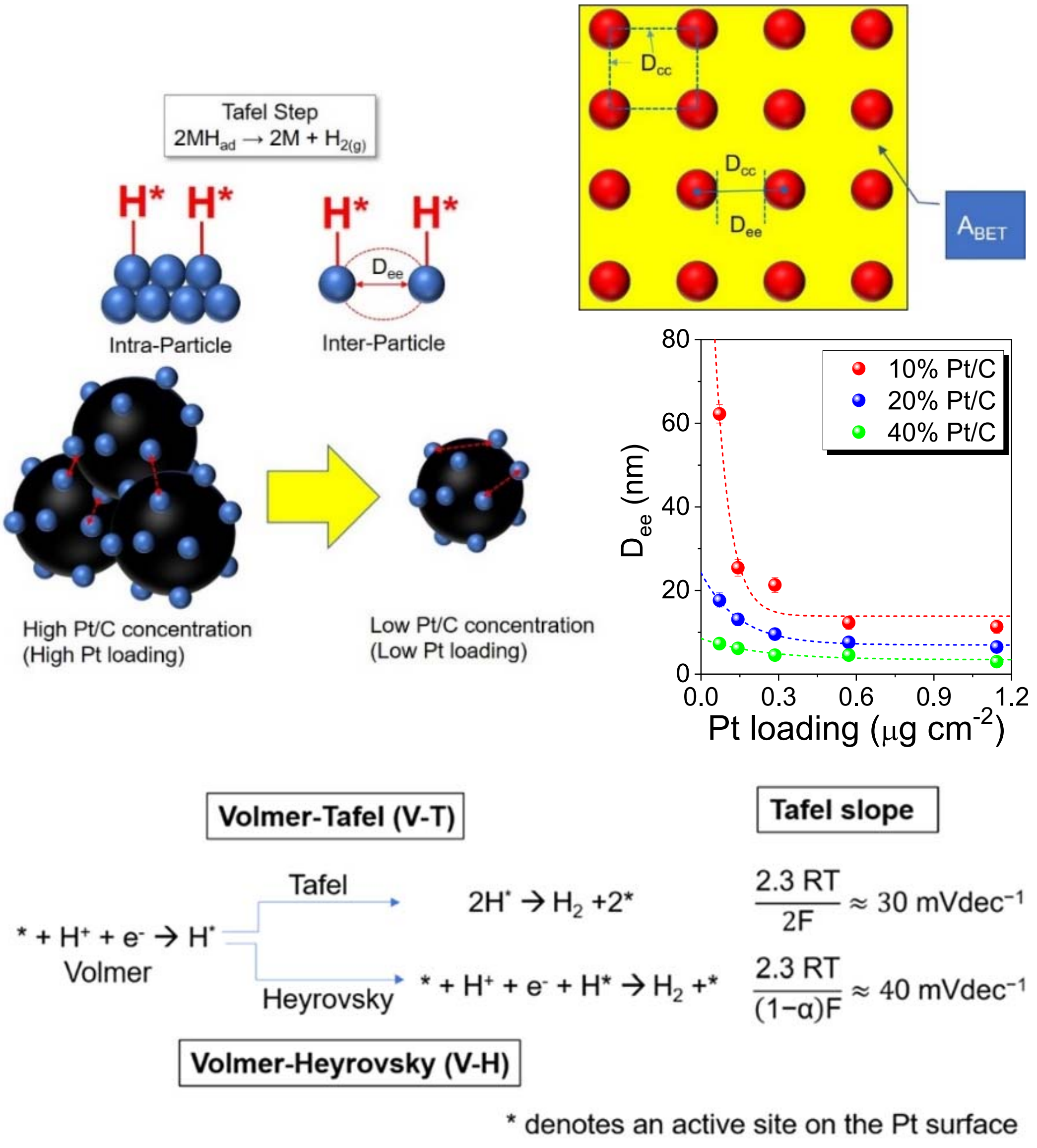

Scheme S1 The electrochemical process evaluated D_{ee} vs. Pt loading; D_{ee} influences the bond formation between two H*, and the HER mechanism.

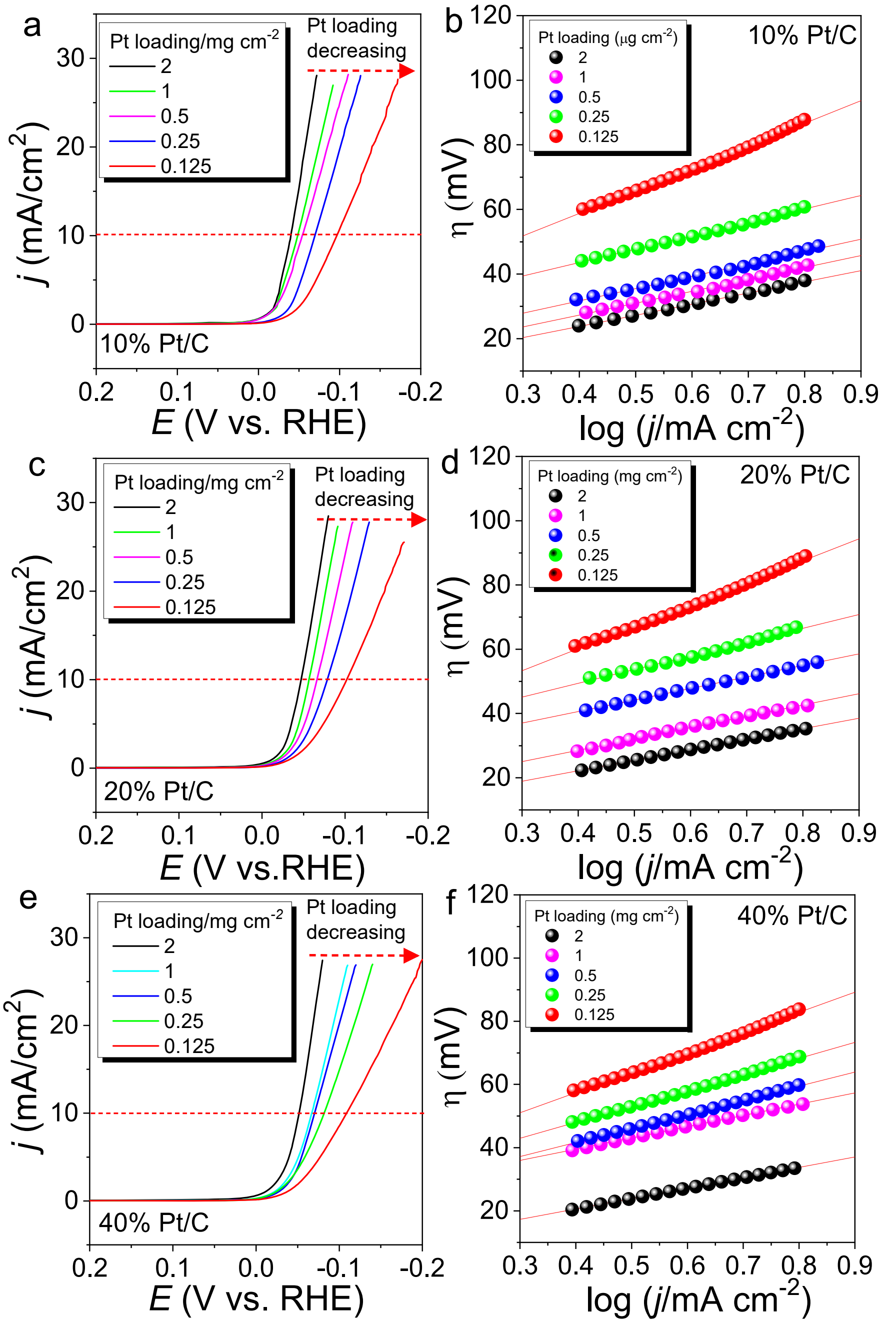

Figure S1 (a)(c)(e) Polarization curves of Pt_m/C with various Pt loadings for HER; (b)(d)(f) The related Tafel plots.

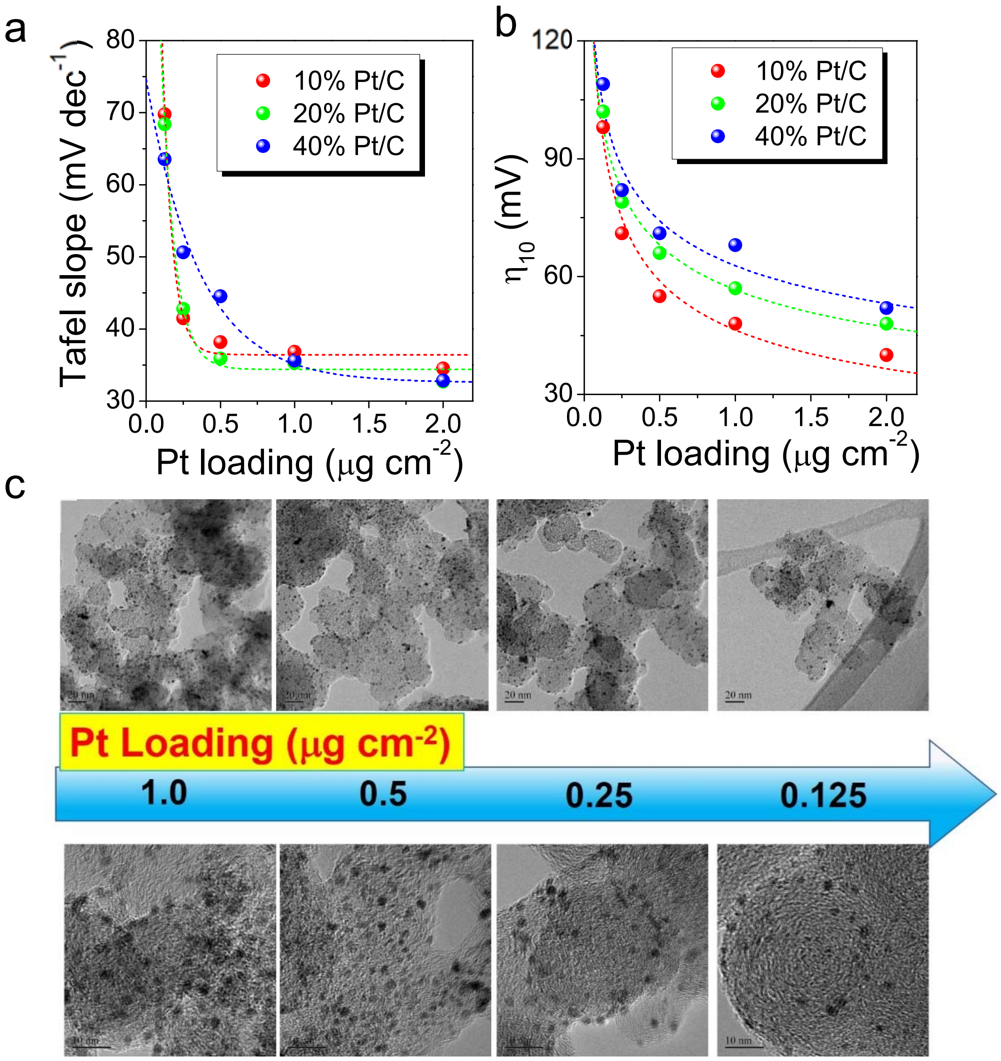

Figure S2 (a) Tafel slopes and b) η_{10}s of Pt_m/C various with Pt loading on the electrode; c) HR-TEM images of Pt_m/C with various Pt loadings in low magnification (above) and high magnification (below).
.

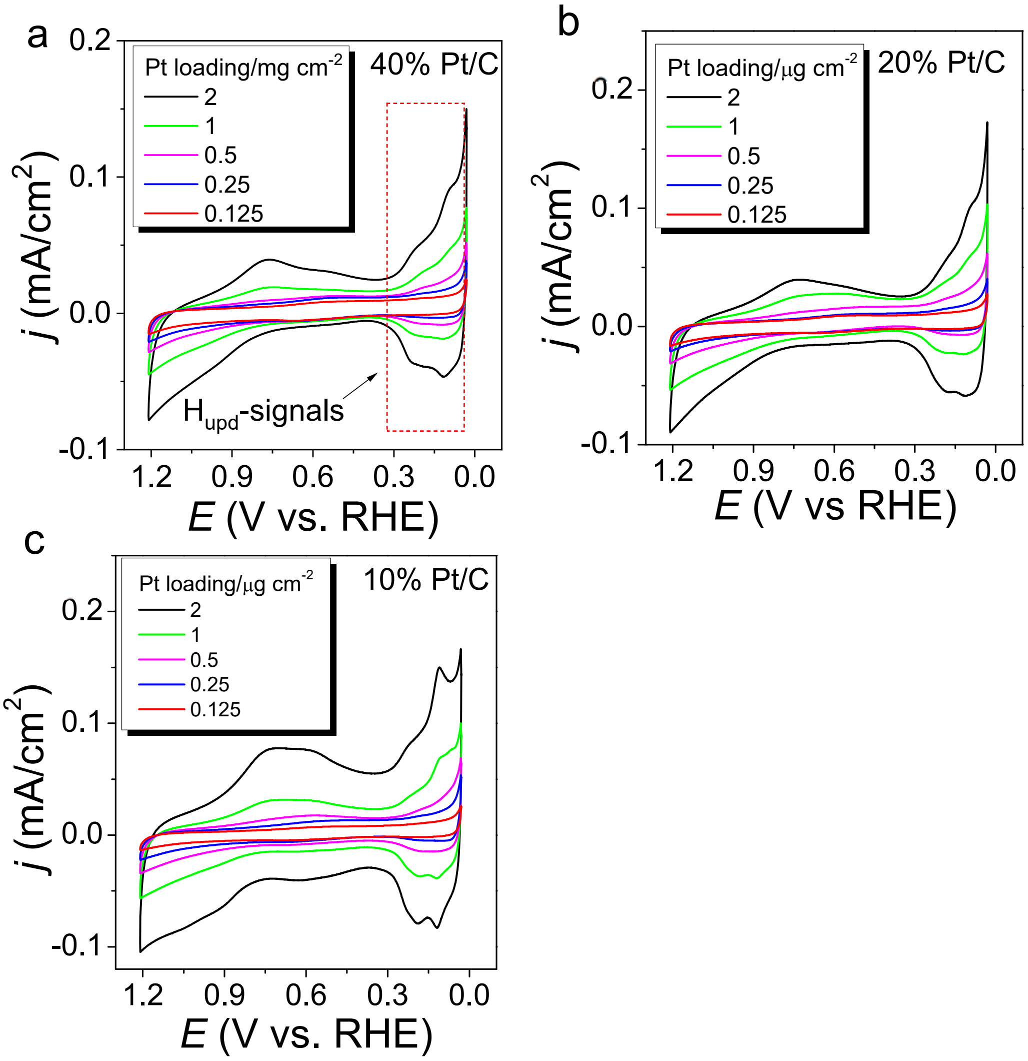

Figure S3 CVs of the Pt_m/C/GCE in Ar-saturated 0.5 M H_2SO_{4aq} at a scan rate of 50 mVs^{-1}.

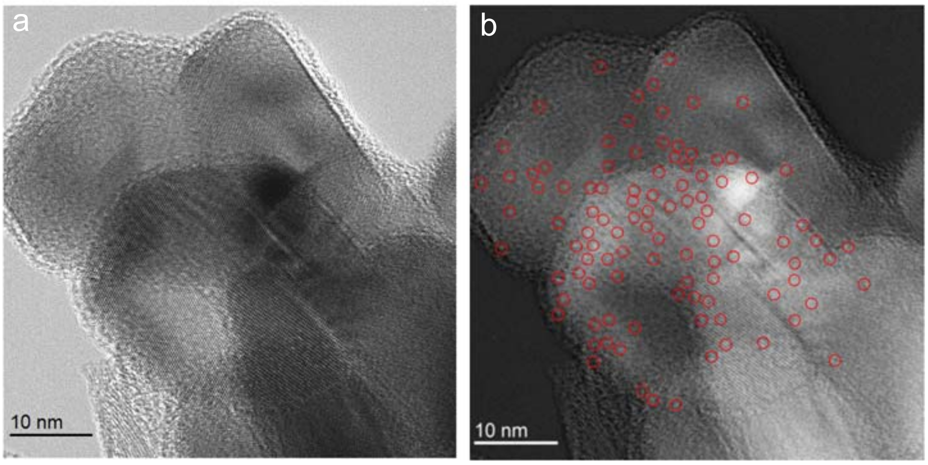

Figure S4 (a) a HR-TEM image of C+RuO_2(Pt^{2+}); (b) a HAADF-STEM image of (a) (aPts are high lighted by red circles).

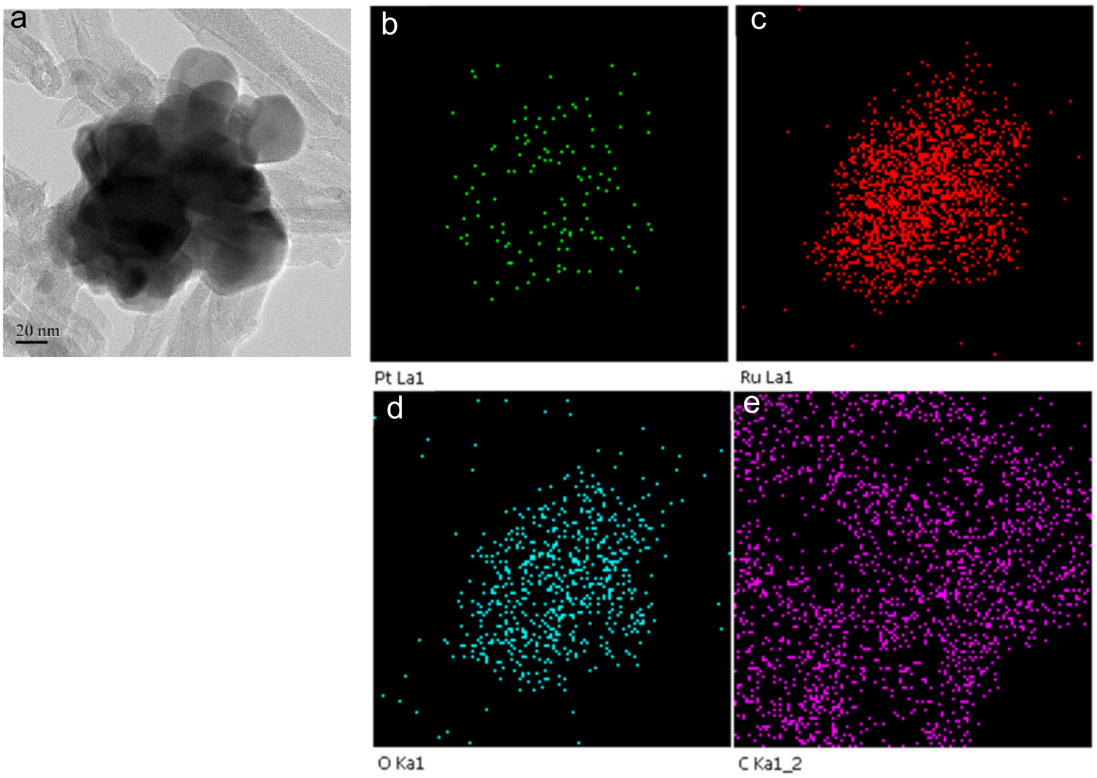

Figure S5 (a) The TEM image of C+RuO_2(Pt^{2+}); (b) Pt (c) Ru (d) O (e) C EDS mapping in (a).

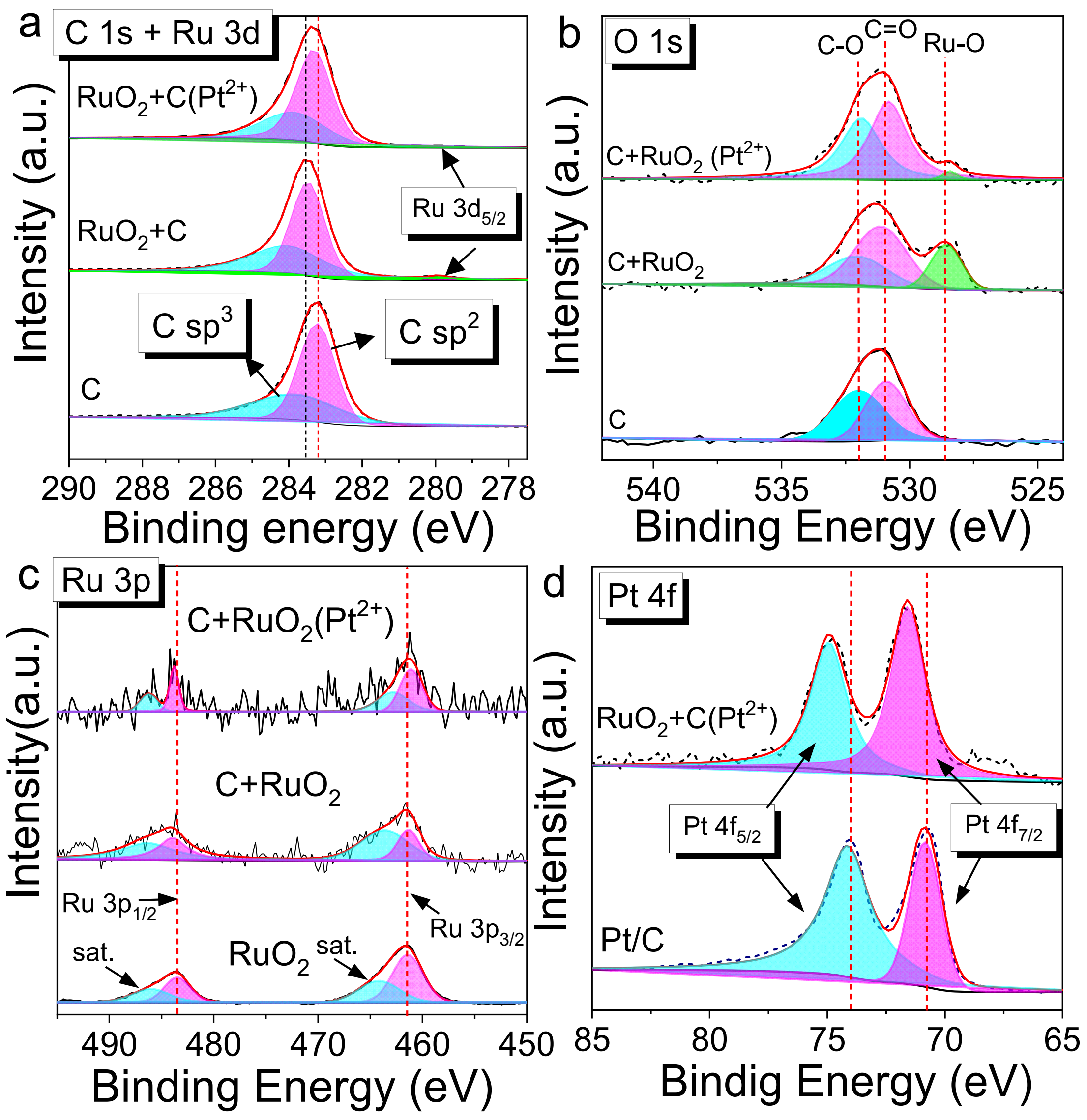

Figure S6 High-resolution XPS (a) C 1s and Ru 3d; (b) O 1s; (c) Ru 3p; (d) Pt 4f spectra of $C+RuO_2(Pt^{2+})$, $C+RuO_2$, C and Pt/C.

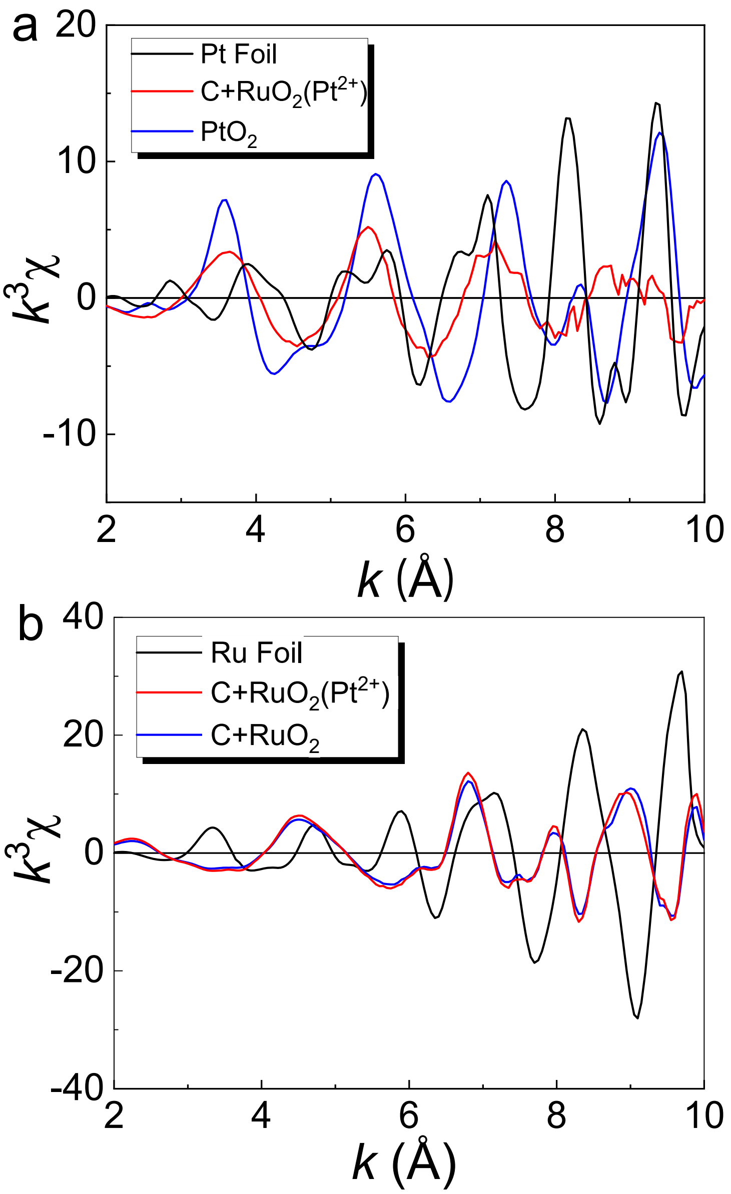

Figure S7 k^3-weighted FT-EXAFS spectra of C+RuO_2, C+RuO_2(Pt^{2+}), Pt foil, PtO_2, and Ru foil at (a) the Pt L_3-edge and (b) the Ru K-edge.

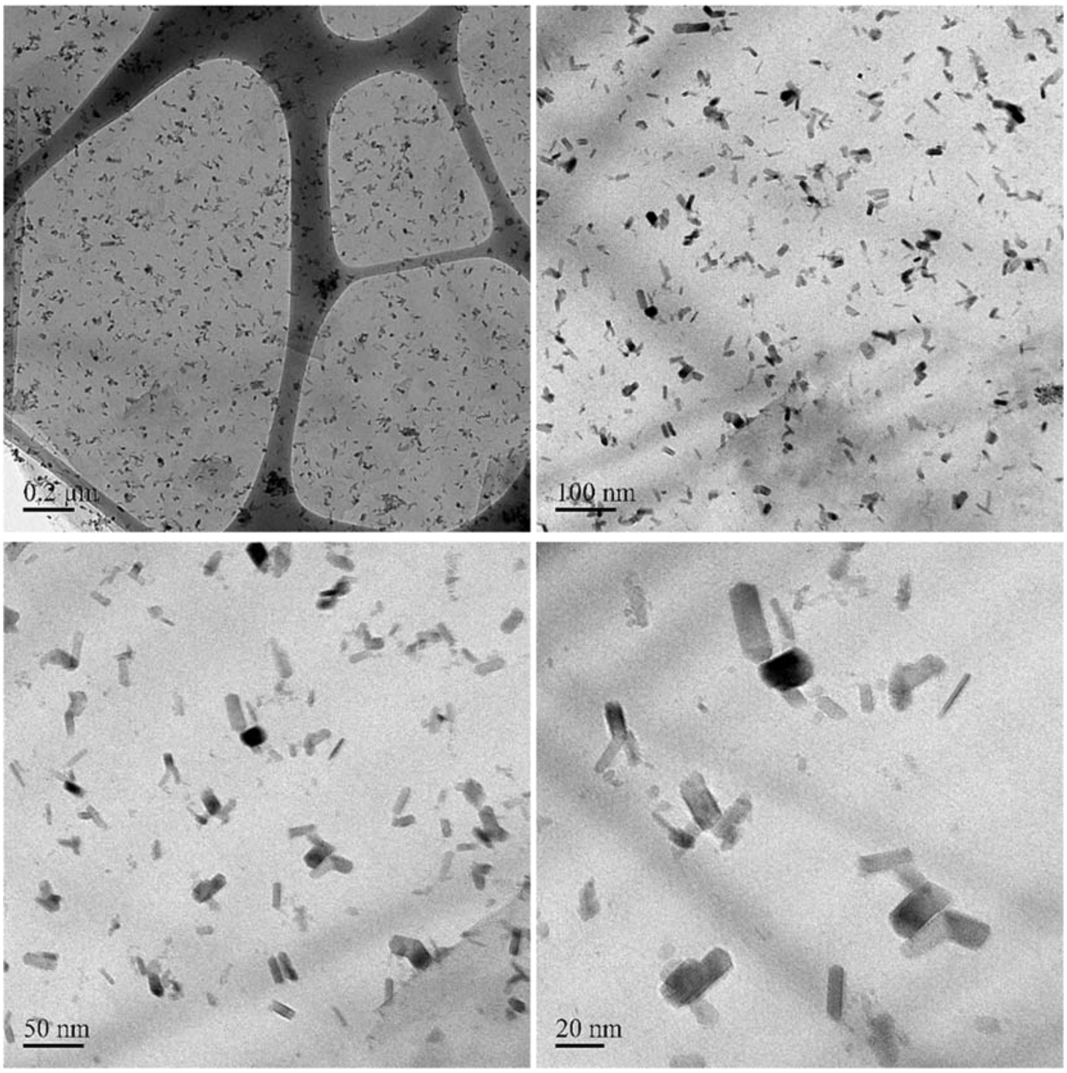

Figure S8 TEM images of RuO_{2NR}/GE.

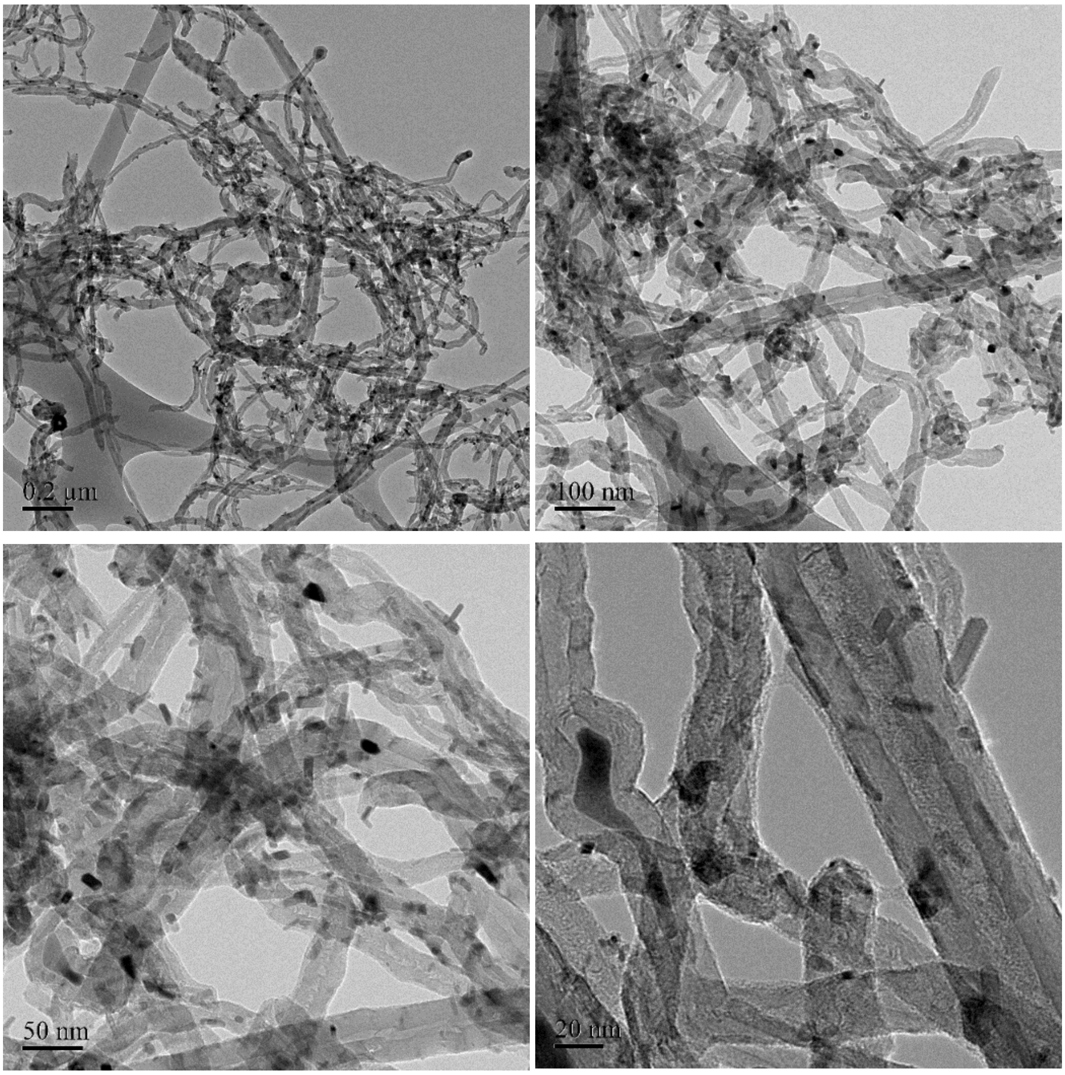

Figure S9 TEM images of RuO_{2NR}/MWCNT.

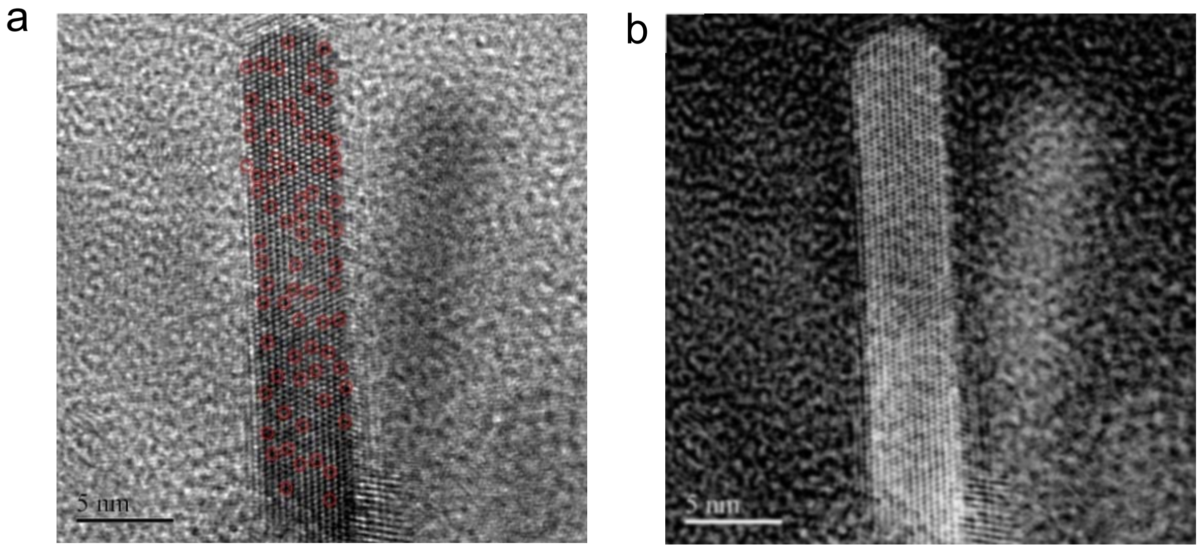

Figure S10 (a) The HR-TEM images of aPt/RuO_{2NR}/GE (ultra-small dark dots are aPts marked by red cycles); (b) The corresponding HAADF-STEM images (ultra-small bright dots are aPts).

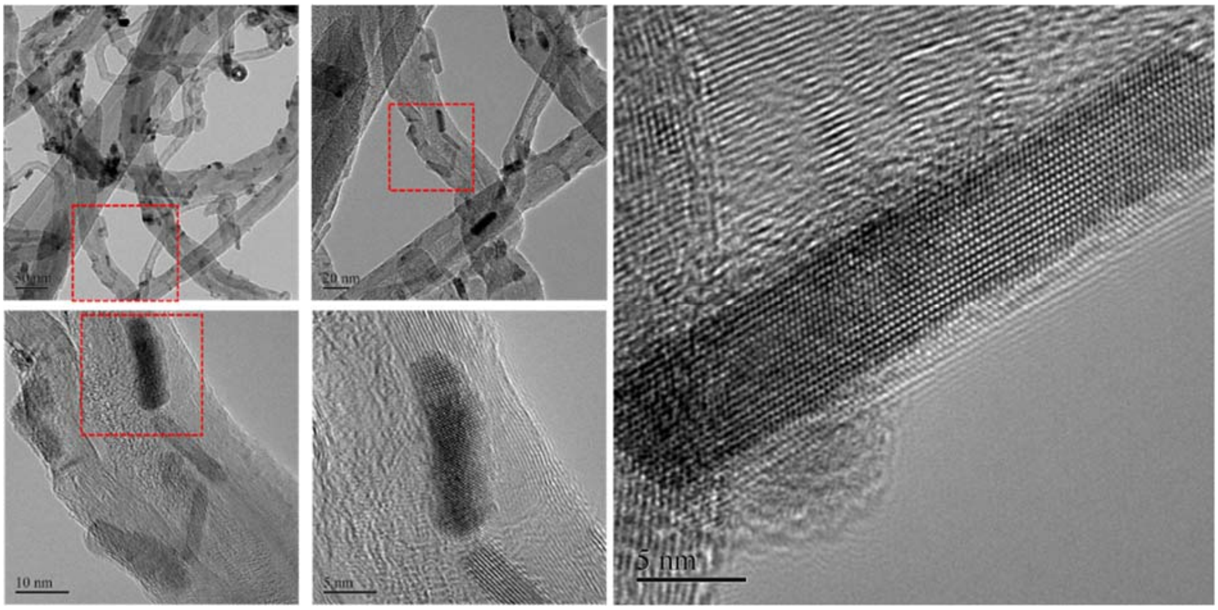

Figure S11 HR-TEM images of aPt/RuO_{2NR}/MWCNT.

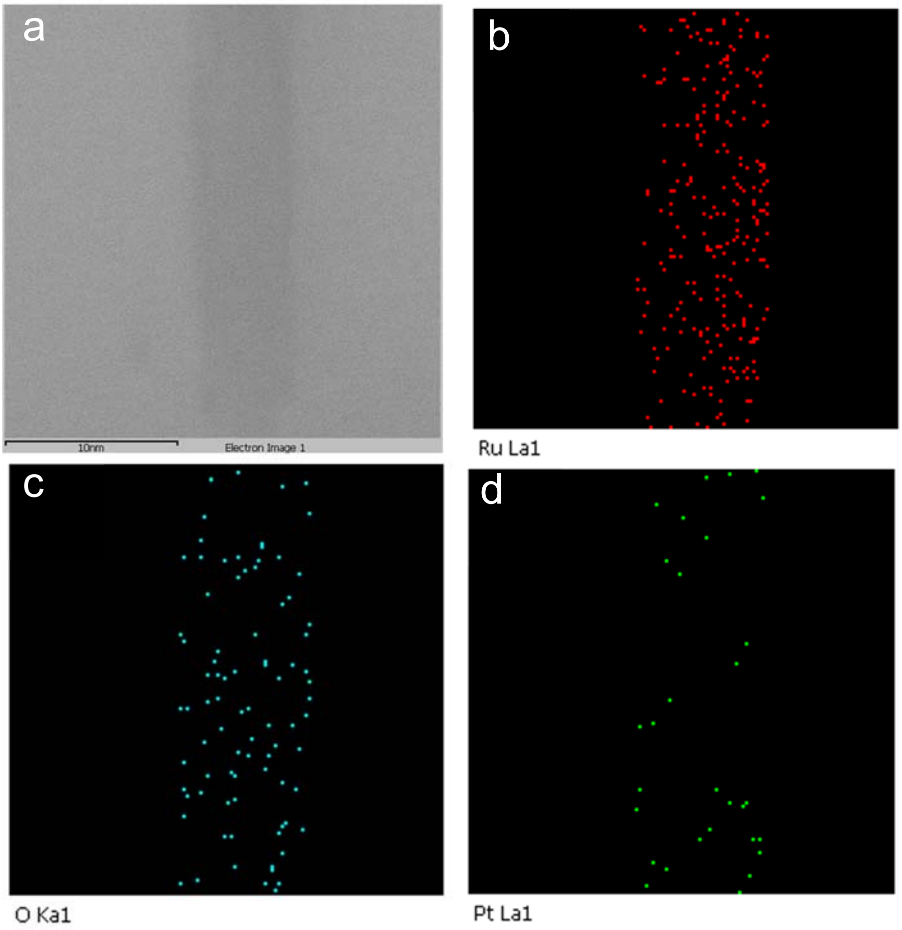

Figure S12 (a) The TEM image of aPt/RuO_{2NR}/GE; (b) Ru (c) O (d) Pt EDS mapping in (a).

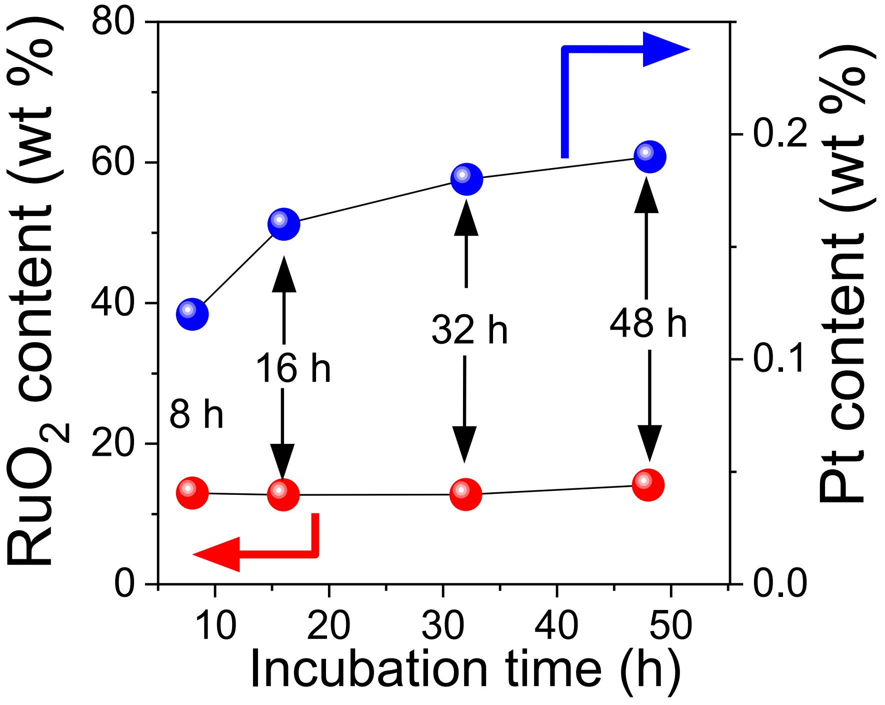

Figure S13 RuO_2 and Pt content in aPt/RuO_{2NR}/Carbon as a function of incubation time in Pt_{aq}.

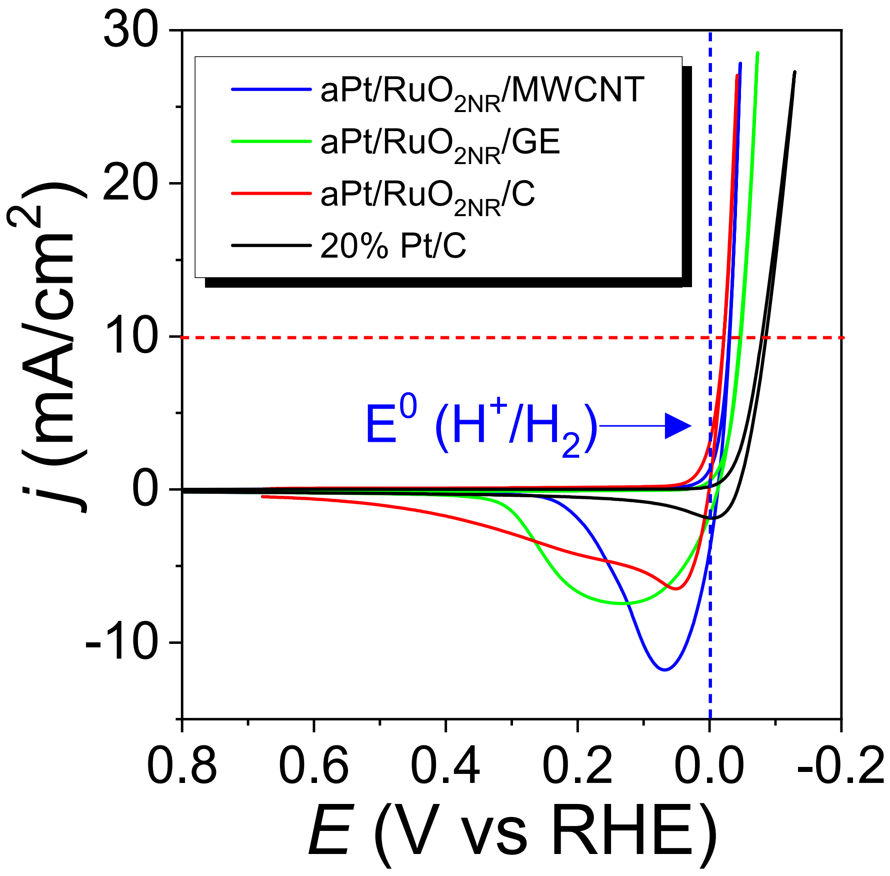

Figure S14 CVs of aPt/RuO_{2NR}/MWCNT, aPt/RuO_{2NR}/GE, aPt/RuO_{2NR}/C, and Pt/C in Ar-saturated 0.5 M H_2SO_{4aq} at a scan rate of 50 mVs^{-1}.

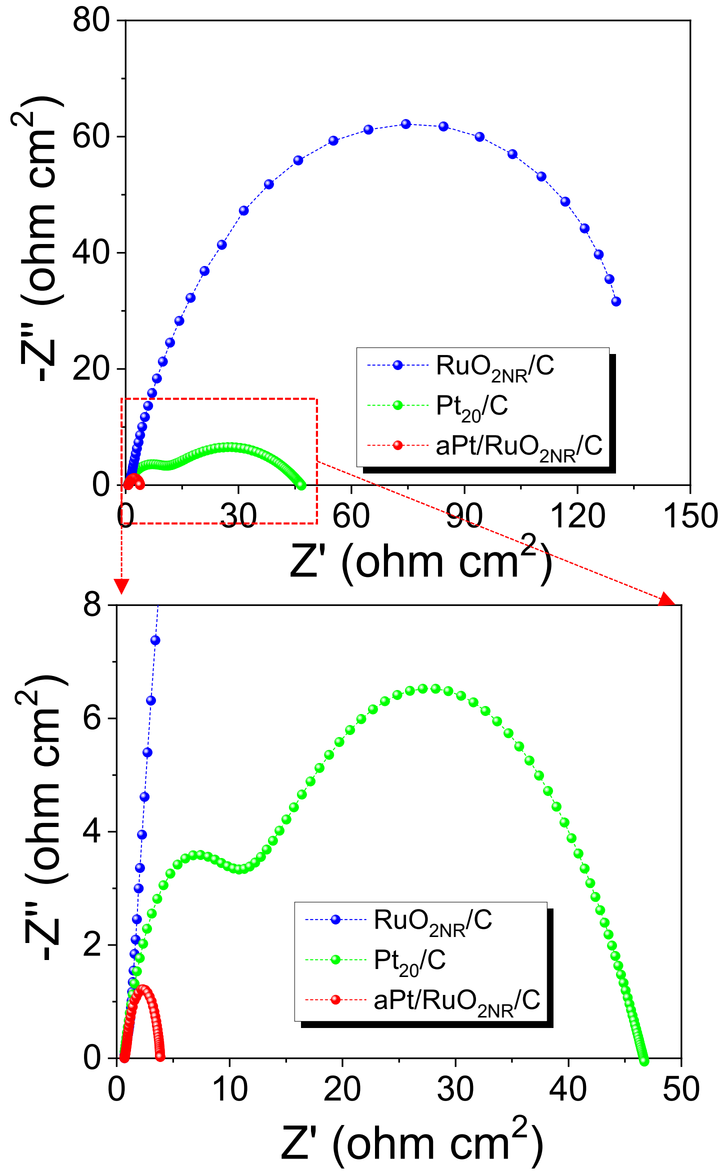

Figure S15 EIS recorded in Ar-purged H_2SO_{4aq} at η = 20 mV.

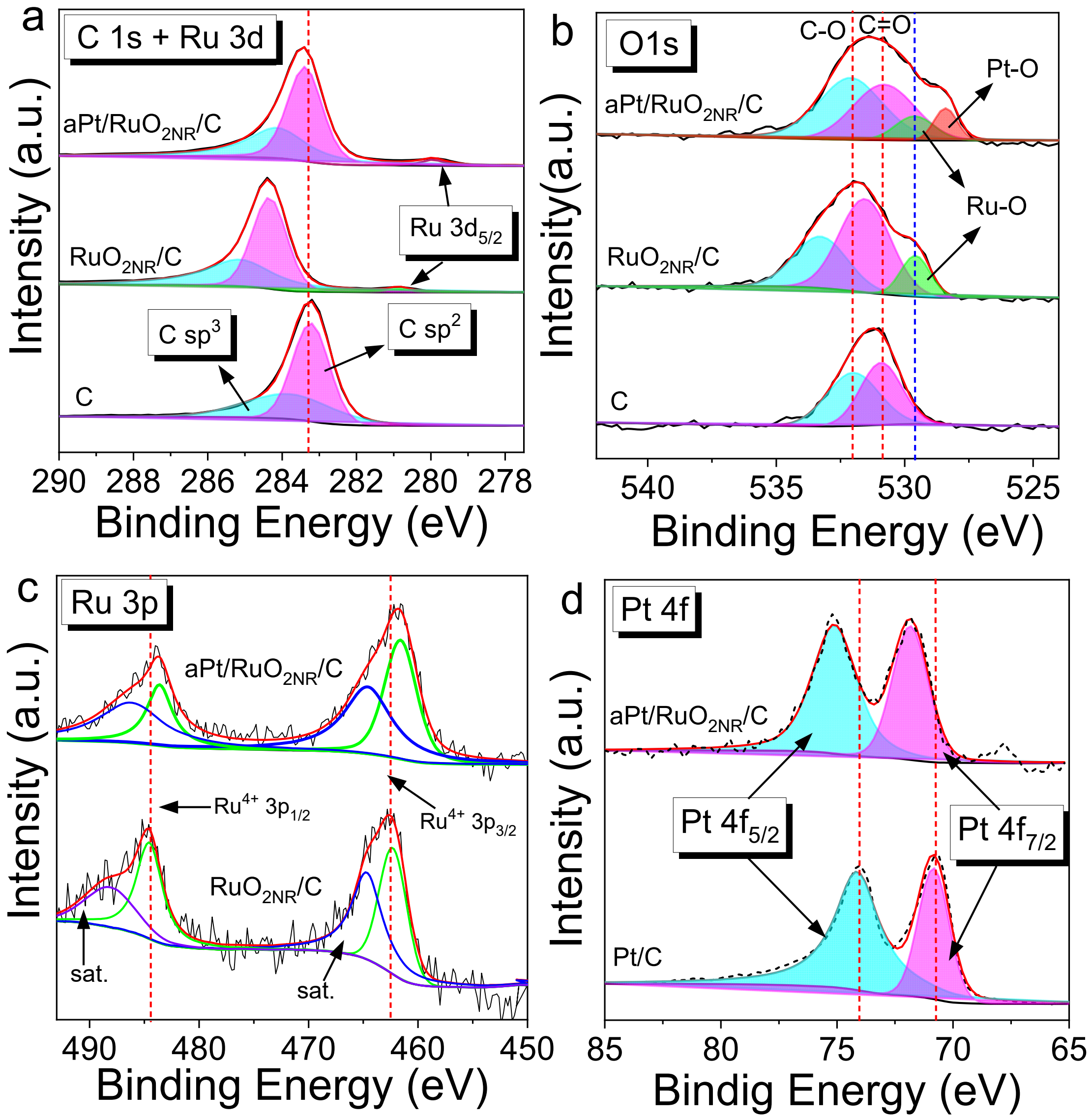

Figure S16 High-resolution XPS (a) C 1s and Ru 3d; (b) O 1s; (c) Ru 3p; (d) Pt 4f spectra of aPt/RuO_{2NR}/C, RuO_{2NR}/C, C and Pt/C.

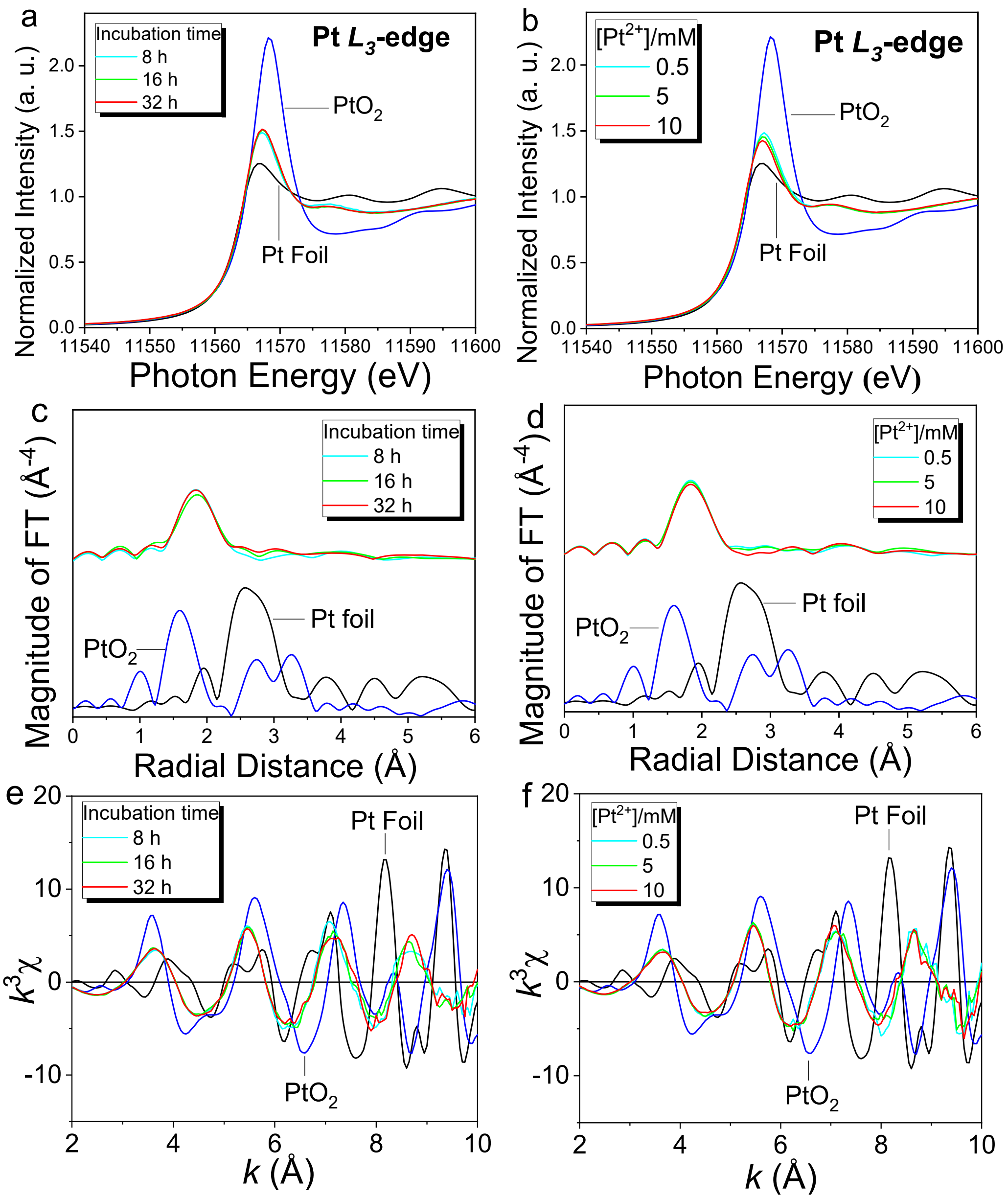

Figure S17 (a)(b) Pt L_3-edge XANES spectra, (c)(d) FT-EXAFS oscillations, and (e)(f) k^3-weighted FT-EXAFS spectra of aPt/RuO_{2NR}/C; (a)(c)(e) for various incubation time in 0.5 mM Pt_{aq} and (b)(d)(f) for incubation time 8 h in various Pt^{2+} concentrations.

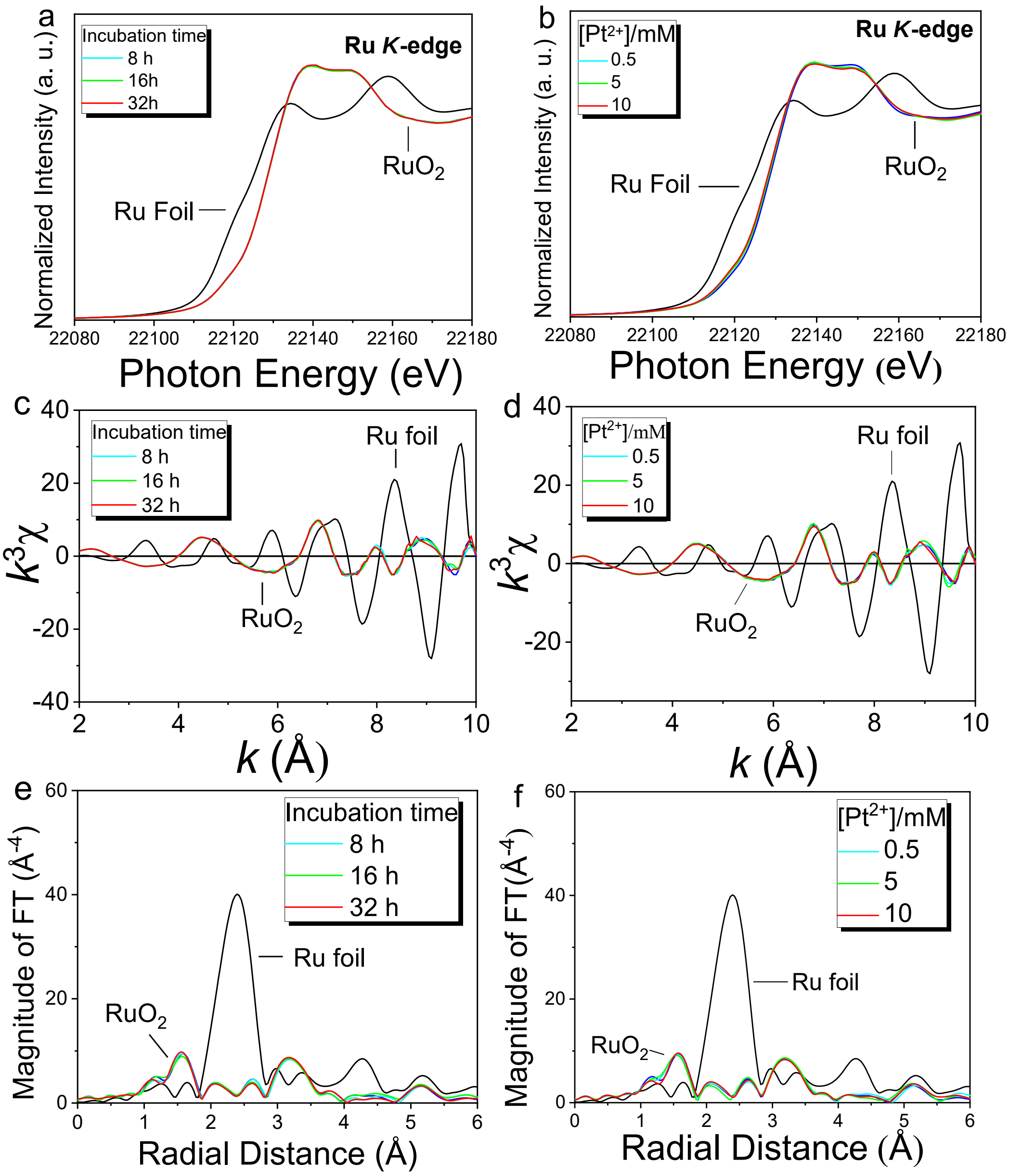

Figure S18 (a)(b) Ru K-edge XANES spectra, (c)(d) FT-EXAFS oscillations, and (e)(f) k^3-weighted FT-EXAFS spectra of aPt/RuO_{2NR}/C; (a)(c)(e) for various incubation time in 0.5 mM Pt_{aq} and (b)(d)(f) for incubation time 8 h in various [Pt^{2+}].

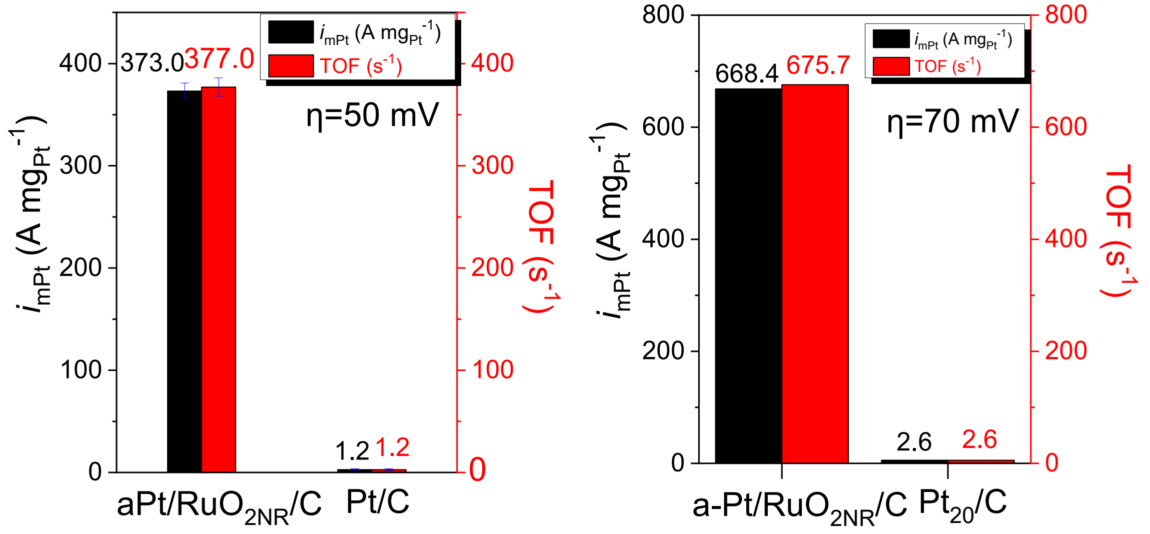

Figure S19 i_{mPt}/TOF for aPt/RuO_{2NR}/C and Pt/C at η of 50 mV and 70 mV.

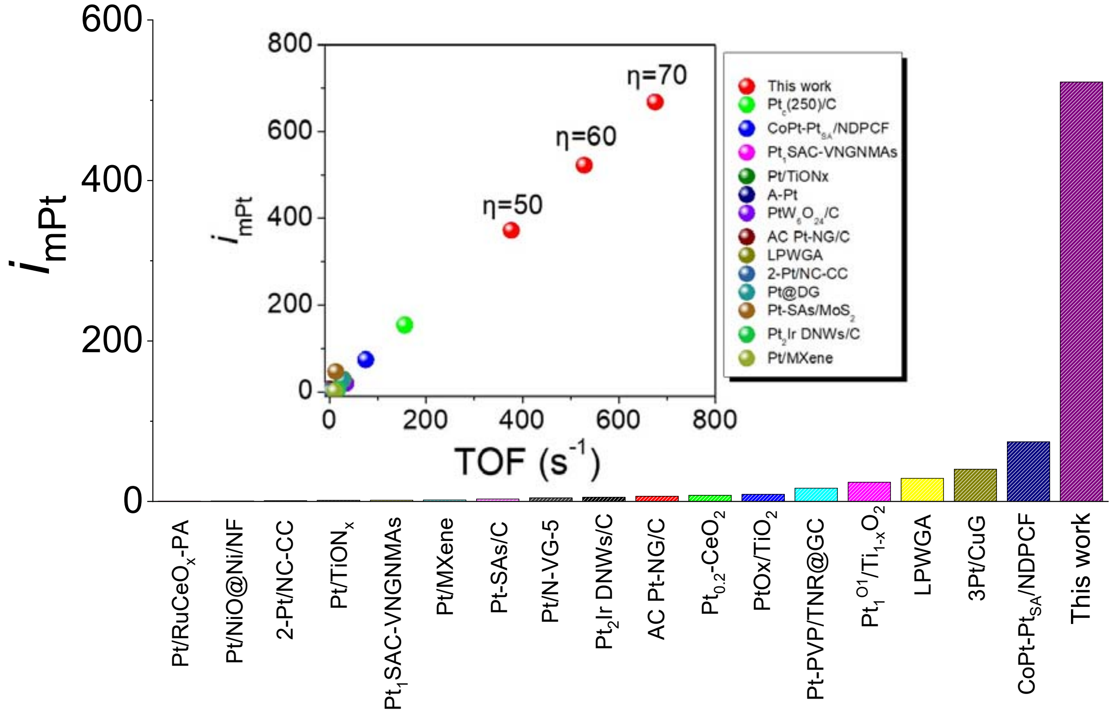

Figure S20 i_{mPt} for the modern Pt-SACs (inset: i_{mPt} vs. TOF for the state-of-the-art Pt-SACs).

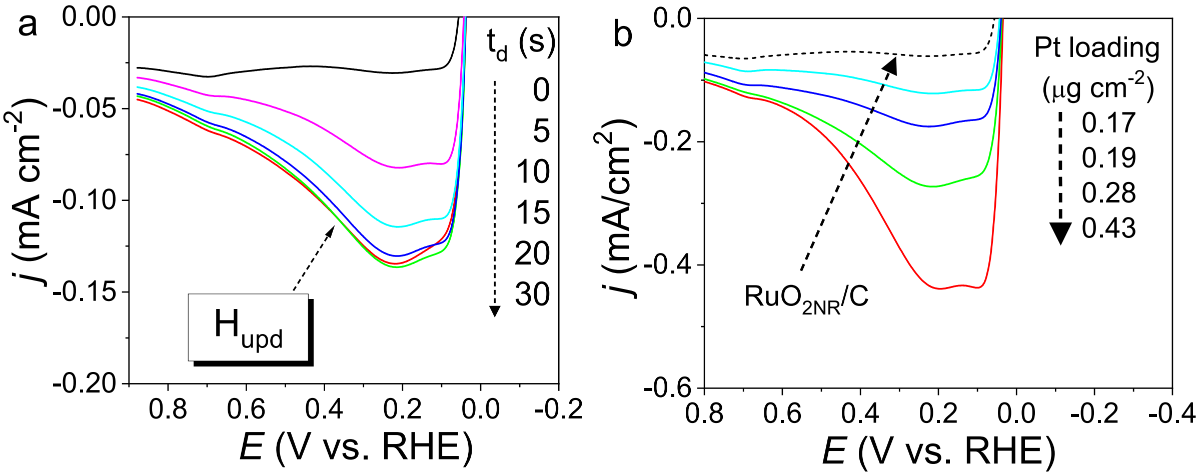

Figure S21 (a) Anodic LSVs for H_{upd} were recorded on aPt/RuO_{2NR}/C (Pt loading is 0.19 μg cm^{-2}) after holding at 0.04 V for various t_d (b) Anodic LSVs for H_{upd} were recorded on aPt/RuO_{2NR}/C with various Pt loading after holding at 0.04 V for 15 s

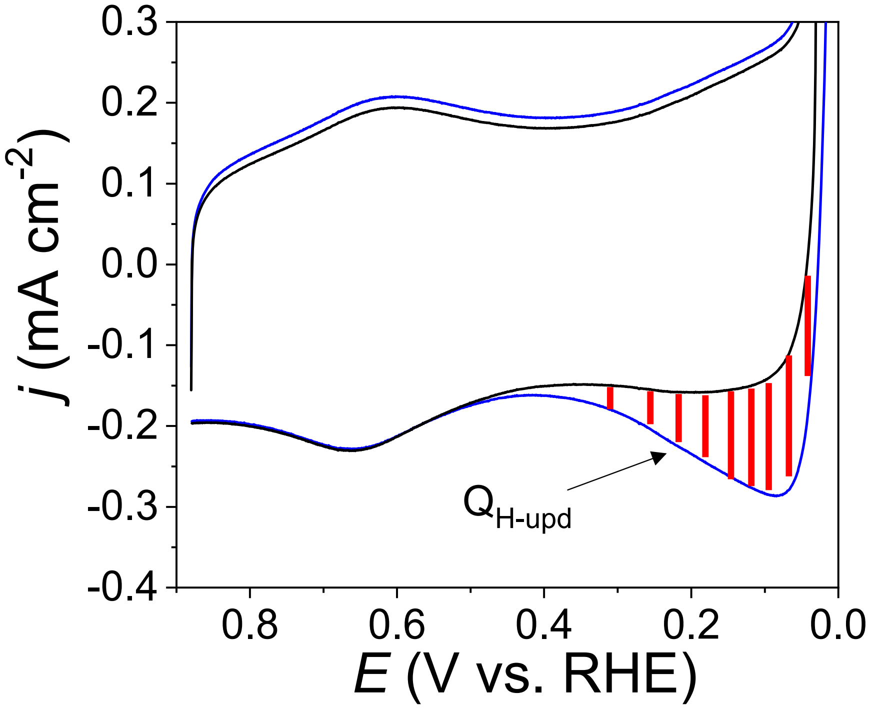

Figure S22 The charge density for desorption of H_{upd} ($Q_{H\text{-}upd}$) from the marked area which is the difference between CV with H_{upd} and CV without H_{upd}.

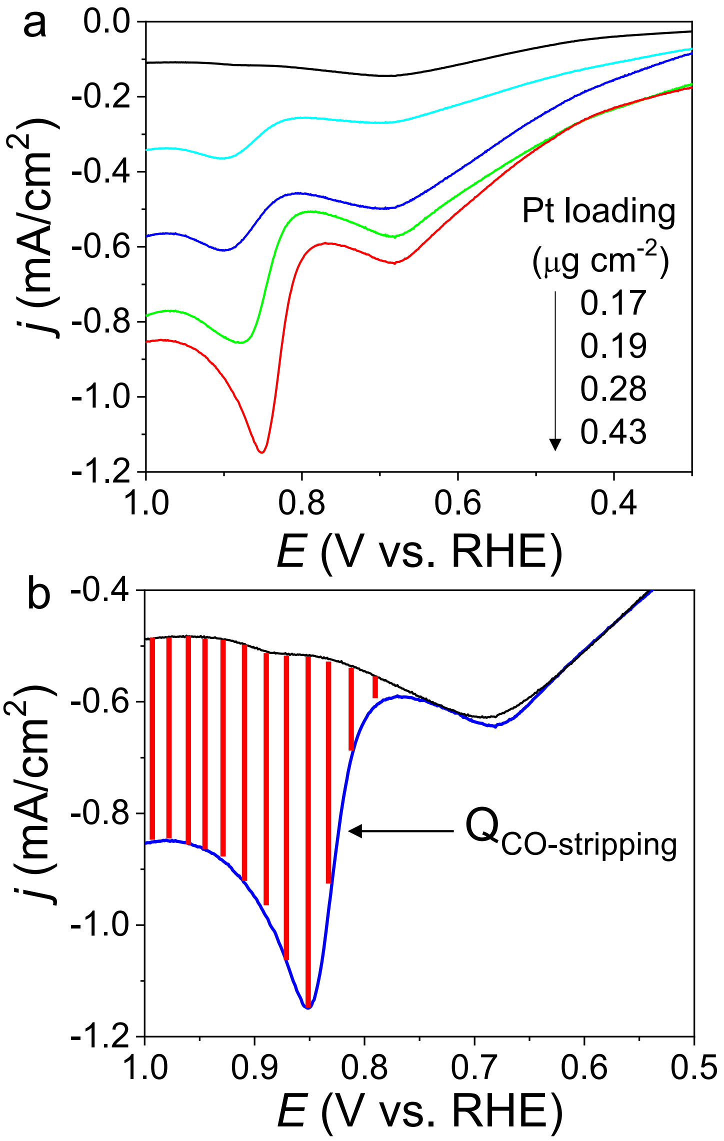

Figure S23 (a) CO stripping curves on aPt/RuO_2/C with various Pt loadings; (b) the charge density for CO-stripping ($Q_{CO\text{-}stripping}$) in the marked area from the difference between CV with CO adsorption and CV without CO adsorption.

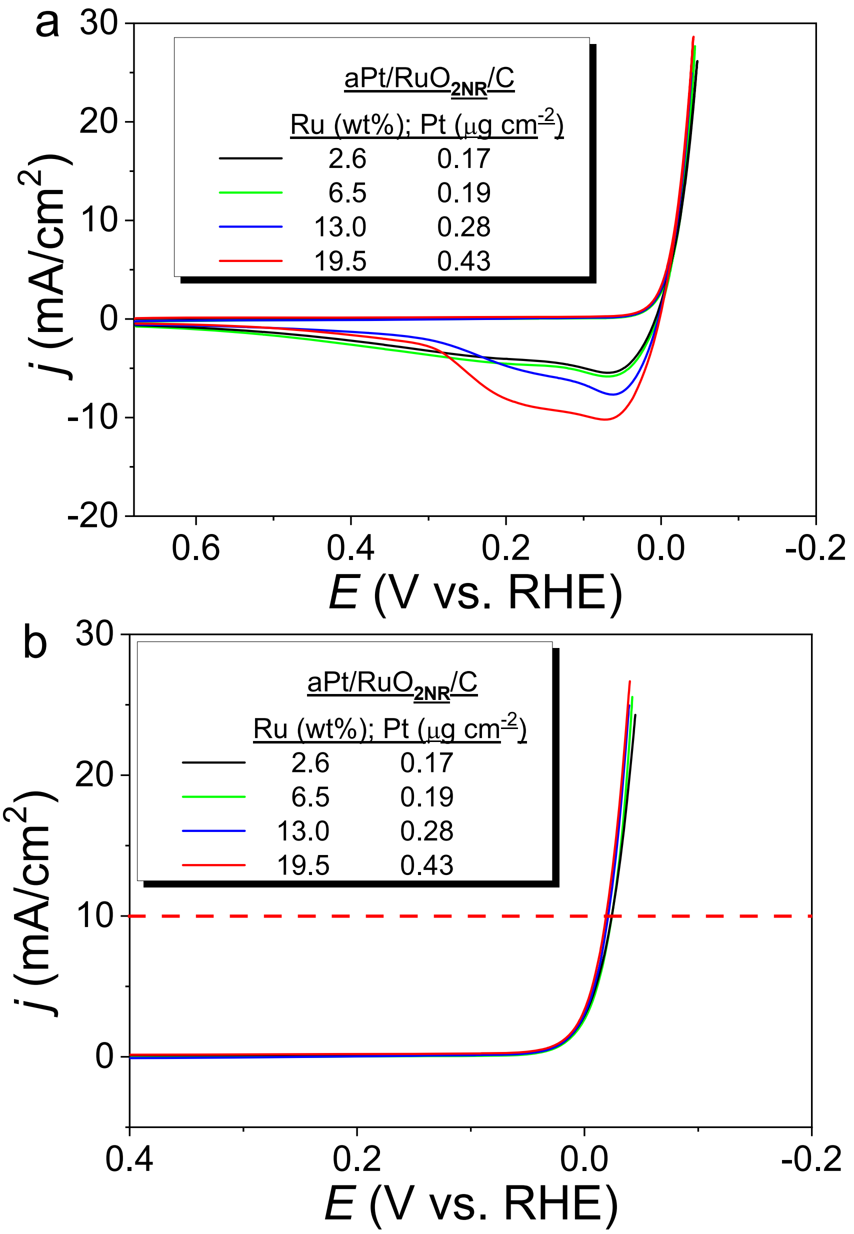

Figure S24 (a) HER polarization curves and associated (b) Tafel plots were recorded in various Pt loadings (from 0.17 to 0.43 μg cm^{-2}) on the HER of aPt/RuO_{2NR}/C.

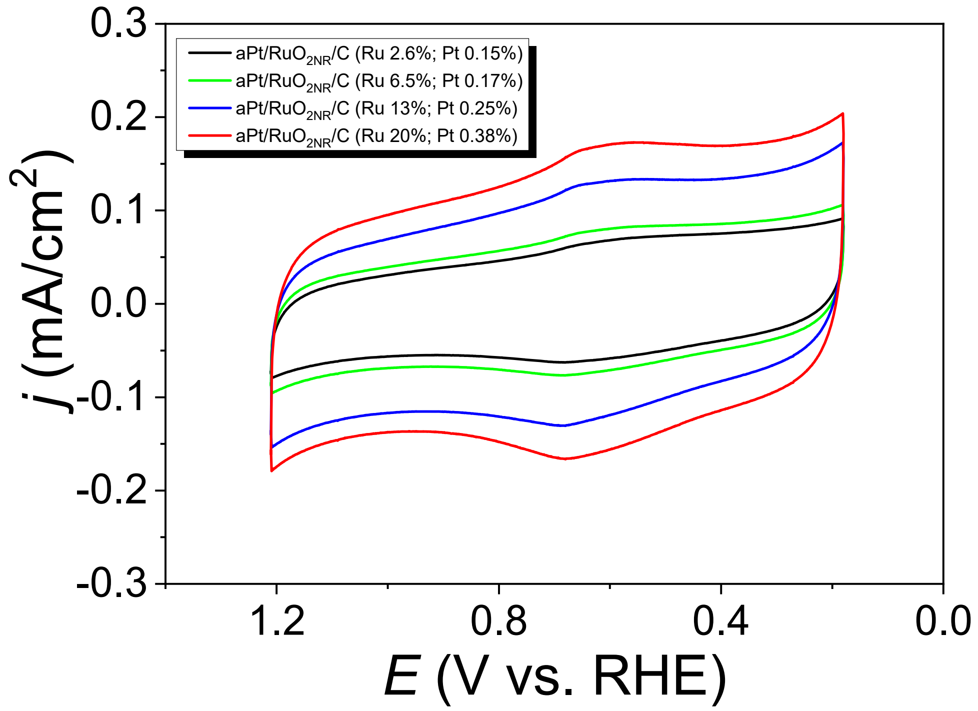

Figure S25 Tracking RuO_2 content by CVs of aPt/RuO_{2NR}/C recorded in the non-Faradaic potential range (electrochemical double layer).

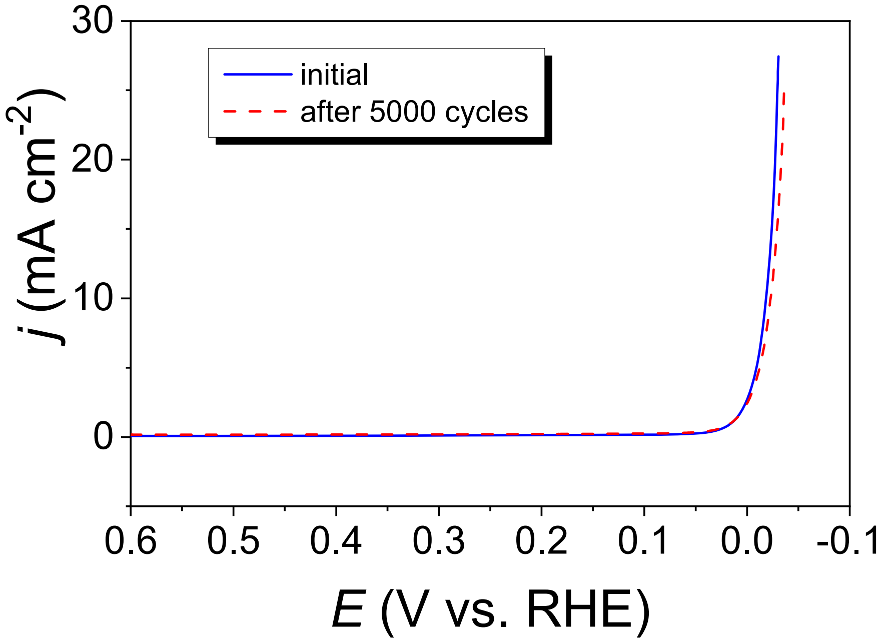

Figure S26 Stability tests of aPt/RuO_{2NR}/Carbon in HER through the CV scanning: the polarization curves before and after 5000 potential cycles are displayed.

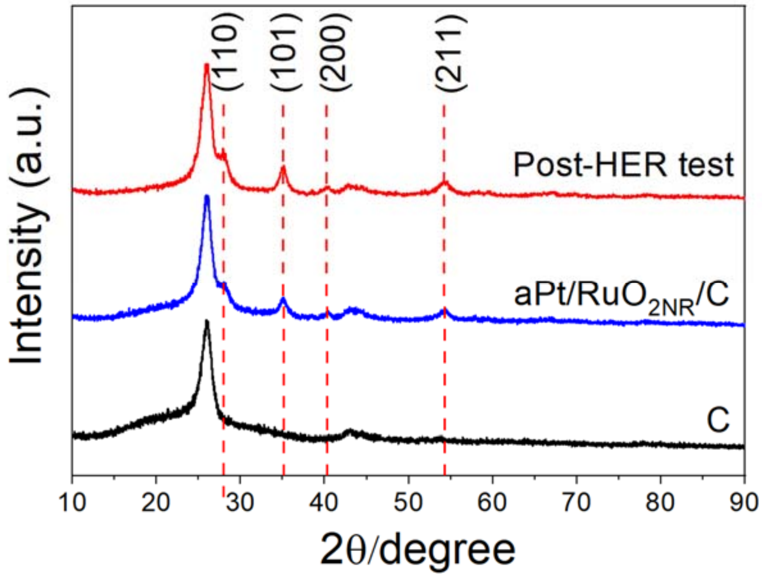

Figure S27 Stability tests of aPt/RuO_{2NR}/Carbon in HER through the XRD analysis: XRD patterns (Cu Kα) of (black line) C and aPt/RuO_{2NR}/C (blue line) before and (red line) after 5000 potential cycles are displayed.

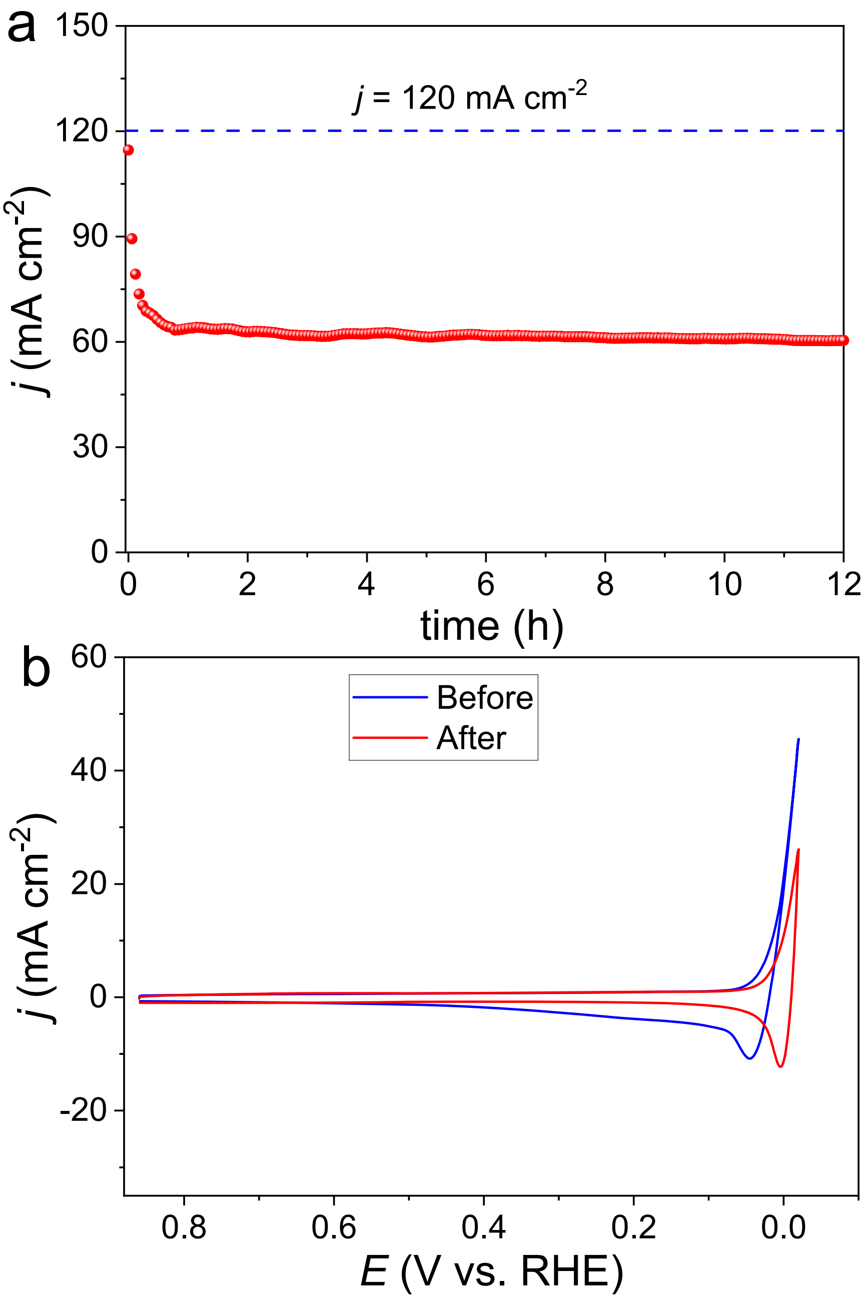

Figure S28. (a) Long-term stability of aPt/RuO_{2NR}/Carbon examined by chronoamperometry at overpotentials of 100 mV (current density ~120 mA cm^{-2}) for HER in 0.5 M H_2SO_{4aq}. (b) CVs of aPt/RuO_{2NR}/Carbon (blue line) before and (red line) after the long-term stability in (a).

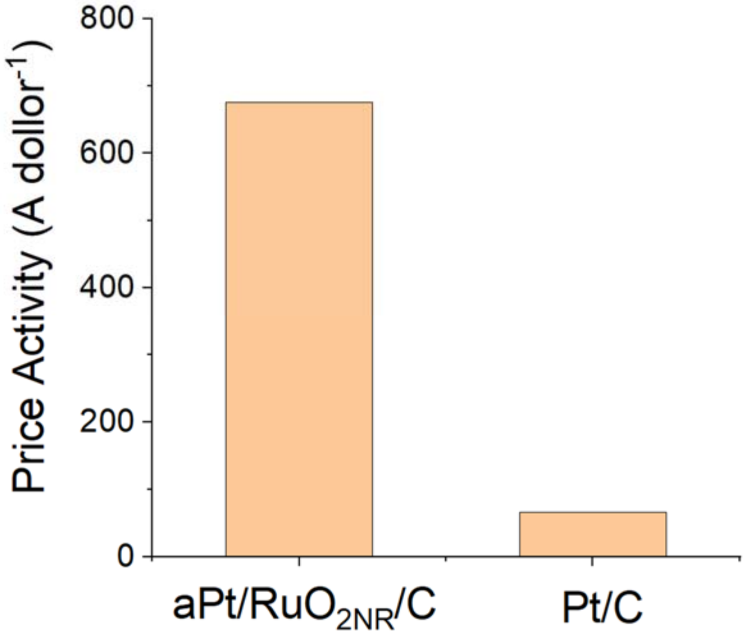

Figure S29 Price activities of aPt/RuO_{2NR}/C and Pt/C at a η of 60 mV. (price activities estimation based on the prices of Ru ($14,146/Kg) and Pt ($28,932/Kg) as found on a website dated 3/21/2024.)

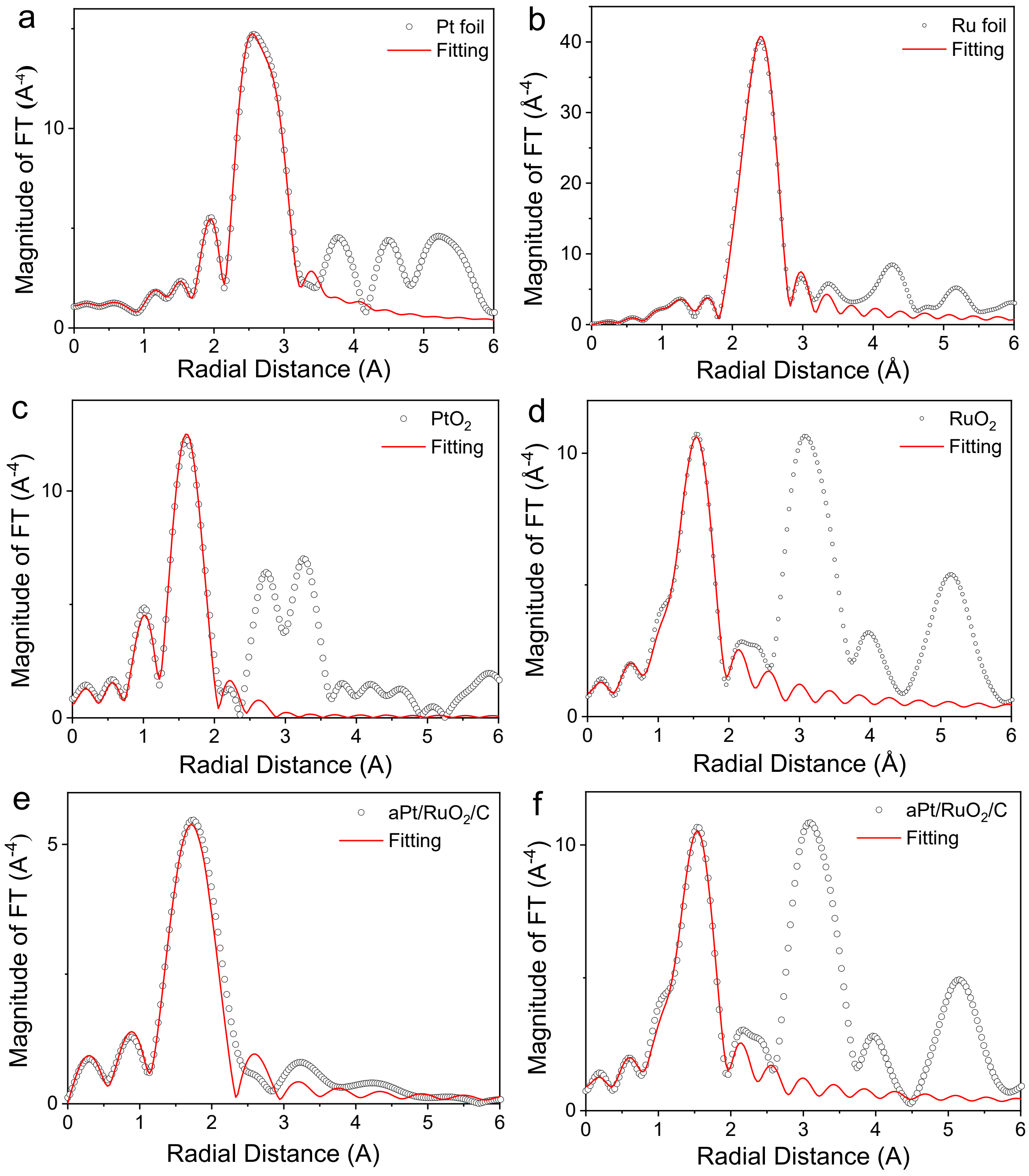

Figure S30. (a)(c)(e) Pt L3-edge and (b)(d)(f) Ru K-edge FT-EXAFS oscillations of Pt foil, PtO_2, Ru foil, RuO_2 and aPt/RuO_2/C catalyst.

Table S1. The best-fit FT-EXAFS parameters of $aPt/RuO_2/C$ catalysts

Sample	Scattering Path	N	R (Å)	σ^2 (Å^2)	R_f
Pt foil	Pt-Pt	12	2.76	0.004	0.003
PtO_2	Pt-O	6	2.02	0.003	0.007
$aPt/RuO_2/C$	Pt-O	3.88	2.09	0.004	0.015
$aPt/RuO_2/C$	Ru-O	6	1.98	0.003	0.005
Ru foil	Ru-Ru	12	2.68	0.004	0.003
RuO_2	Ru-O	6	1.98	0.003	0.003

Table S2. HER performances of Pt-SACs and Pt-catalysts

Catalysts	Tafel (mV/dec)	η_{10} (mV)	TOF (s^{-1})	$i_{mPt(\eta/mV)}$ (A/mg)	electrolyte	Refs.
$aPt/RuO_{2NR}/C$	30.2	18	377.0_{50}	373.0_{50}	0.5M H_2SO_4	This work
$aPt/RuO_{2NR}/C$	30.2	18	528.4_{60}	522.7_{60}	0.5M H_2SO_4	
$aPt/RuO_{2NR}/C$	30.2	18	675.7_{70}	668.4_{70}	0.5M H_2SO_4	
$Pt_c(250)/C$	30.9	37.4	156.1_{60}	154.5_{60}	0.5M H_2SO_4	s[1]
CoPt-Pt_{SA}/NDPCF	24.84	20	75.11_{50}	74.31_{50}	0.5M H_2SO_4	s[7]
Pt/N-VG-5	52.2	42	N/A	4.45_{50}	0.5M H_2SO_4	s[8]
Pt_1 SAC-VNGNMAs	49	15	$\sim1.5_{50}$	$\sim1.5_{50}$	0.5M H_2SO_4	s[9]
$Pt/TiON_x$	29.5	N/A	12.15_{50}	1.41_{50}	0.1 M $HClO_4$	s[10]
A-Pt	97.0	89.7	0.487_{10}	0.948_{10}	0.5M H_2SO_4	s[11]
PtW_6O_{24}/C	29.8	22	33.35_{100}	20.175_{77}	0.5M H_2SO_4	s[12]
$Pt_1{}^{O1}/Ti_{1-x}O_2$	31	22	N/A	23.92_{50}	0.5M H_2SO_4	s[13]
Pt-PVP/TNR@GC	27	21	N/A	16.53_{50}	0.5M H_2SO_4	s[14]
AC Pt-NG/C	27	35.28	0.0927_{50}	6.508_{50}	0.5M H_2SO_4	s[15]
$Pt/RuCeO_x$-PA	31	41	N/A	0.375_{50}	0.5M H_2SO_4	s[16]
3Pt/CuG	27.6	20	N/A	40.1_{50}	0.5M H_2SO_4	s[17]
LPWGA	30	42	29.05_{50}	28.70_{50}	0.5M H_2SO_4	s[18]
2-Pt/NC-CC	36.65	123	0.9354_{50}	0.907_{50}	0.5M H_2SO_4	s[19]
Pt@DG	48	30	26.41_{100}	28.38_{100}	0.5M H_2SO_4	s[20]
Pt/NiO@Ni/NF	40	34	N/A	0.532_{50}	1M KOH	s[21]
Pt-SAs/C	43	38	N/A	3.01_{50}	0.5M H_2SO_4	s[22]
$Pt_{0.2}$-CeO_2	35	N/A	N/A	7.6_{50}	0.5M H_2SO_4	s[23]
Pt-SAs/MoS_2	28	32	12.83_{50}	47.48_{100}	0.5M H_2SO_4	s[24]
PtO_x/TiO_2	40	N/A	N/A	8.68_{50}	0.5M H_2SO_4	s[25]
Pt_2Ir DNWs/C	20.9	26	17.08_{50}	5.17_{50}	0.5M H_2SO_4	s[26]
Pt/MXene	297	34	10.66	1.847_{50}	0.5M H_2SO_4	s[27]

References

(S1) Huang, J. F.; Zeng, R. H.; Chen, J. L., Thermostable carbon-supported subnanometer-sized (<1 nm) Pt clusters for the hydrogen evolution reaction. *J. Mater. Chem. A* **2021,** *9*, 21972-21980.

(S2) Shan, J. Q.; Ye, C.; Jiang, Y. L.; Jaroniec, M.; Zheng, Y.; Qiao, S. Z., Metal-metal interactions in correlated single-atom catalysts. *Sci. Adv.* **2022,** *8*, eabo0762.

(S3) Tan, T. L.; Wang, L. L.; Zhang, J.; Johnson, D. D.; Bai, K. W., Platinum Nanoparticle During Electrochemical Hydrogen Evolution: Adsorbate Distribution, Active Reaction Species, and Size Effect. *ACS Catal.* **2015,** *5*, 2376-2383.

(S4) Huang, J. F.; Chen, H. Y., Heat-Assisted Electrodissolution of Platinum in an Ionic Liquid. *Angew. Chem., Int. Ed.* **2012,** *51*, 1684-1688.

(S5) Huang, J. F.; Hsiao, H. Y., Electrochemically Identifying Degradation Pathways of Carbon-Supported Pt Catalysts Assists in Designing Highly Durable Catalysts. *ACS Appl. Mater. Interfaces* **2016,** *8*, 33749-33754.

(S6) Huang, J. F.; Yang, H. W., Electrochemical Quantifying, Counting, and Sizing Supported Pt Nanoparticles in Real Time. *Anal. Chem.* **2016,** *88*, 6403-6409.

(S7) Yang, W. W.; Cheng, P.; Li, Z.; Lin, Y. X.; Li, M. Y.; Zi, J. Z.; Shi, H. H.; Li, G. S.; Lian, Z. C.; Li, H. X., Tuning the Cobalt-Platinum Alloy Regulating Single-Atom Platinum for Highly Efficient Hydrogen Evolution Reaction. *Adv. Funct. Mater.* **2022,** *32*, 202205920.

(S8) Yang, H. Z.; Yang, Z. J.; Han, Z. J.; Chu, D. W.; Chen, C. Q.; Xie, X. Y.; Shang, L.; Zhang, T. R., Highly dispersed platinum deposited on nitrogen-doped vertical graphene array for efficient electrochemical hydrogen evolution. *2D Mater.* **2022,** *9*, 045011.

(S9) Chi, K.; Chen, Z. X.; Xiao, F.; Guo, W.; Xi, W.; Liu, J.; Yan, H.; Zhang, Z. Y.; Xiao, J.; Liu, J.; Luo, J.; Wang, S.; Loh, K. P., Maximizing the utility of single atom electrocatalysts on a 3D graphene nanomesh. *J. Mater. Chem. A* **2019,** *7*, 15575-15579.

(S10) Smiljanic, M.; Panic, S.; Bele, M.; Ruiz-Zepeda, F.; Pavko, L.; Gasparic, L.; Kokalj, A.; Gaberscek, M.; Hodnik, N., Improving the HER Activity and Stability of Pt Nanoparticles by Titanium Oxynitride Support. *ACS Catal.* **2022,** *12*, 13021-13033.

(S11) Ma, Z. H.; Chen, C.; Cui, X. Z.; Zeng, L. M.; Wang, L. J.; Jiang, W.; Shi, J. L., Hydrogen Evolution/Oxidation Electrocatalysts by the Self-Activation of Amorphous Platinum. *ACS Appl. Mater. Interfaces* **2021,** *13*, 44224-44233.

(S12) Yu, F. Y.; Lang, Z. L.; Yin, L. Y.; Feng, K.; Xia, Y. J.; Tan, H. Q.; Zhu, H. T.; Zhong, J.; Kang, Z. H.; Li, Y. G., Pt-O bond as an active site superior to Pt^0 in hydrogen evolution reaction. *Nat. Commun.* **2020,** *11*, 490.

(S13) Lu, F.; Yi, D.; Liu, S. J.; Zhan, F.; Zhou, B.; Gu, L.; Golberg, D.; Wang, X.; Yao, J. N., Engineering Platinum-Oxygen Dual Catalytic Sites via Charge Transfer towards High-Efficient Hydrogen Evolution.

Angew. Chem. Int. Ed. **2020,** *59*, 17712-17718.
(S14) Li, C.; Chen, Z.; Yi, H.; Cao, Y.; Du, L.; Hu, Y. D.; Kong, F. P.; Campen, R. K.; Gao, Y. Z.; Du, C. Y.; Yin, G. P.; Zhang, I. Y.; Tong, Y. J., Polyvinylpyrrolidone-Coordinated Single-Site Platinum Catalyst Exhibits High Activity for Hydrogen Evolution Reaction. *Angew. Chem. Int. Ed.* **2020,** *59*, 15902-15907.
(S15) Sun, M. H.; Ji, J. P.; Hu, M. Y.; Weng, M. Y.; Zhang, Y. P.; Yu, H. S.; Tang, J. J.; Zheng, J. C.; Jiang, Z.; Pan, F.; Liang, C. D.; Lin, Z., Overwhelming the Performance of Single Atoms with Atomic Clusters for Platinum-Catalyzed Hydrogen Evolution. *ACS Catal.* **2019,** *9*, 8213-8223.
(S16) Liu, T. T.; Gao, W. B.; Wang, Q. Q.; Dou, M. L.; Zhang, Z. P.; Wang, F., Selective Loading of Atomic Platinum on a $RuCeO_x$ Support Enables Stable Hydrogen Evolution at High Current Densities. *Angew. Chem. Int. Ed.* **2020,** *59*, 20423-20427.
(S17) Xu, Y. D.; Meng, D. M.; Wang, L. W.; Yang, H.; Guo, X. F.; Zhu, Y.; Wang, T.; Ding, W. P., Subtle modulation on electronic properties of platinum by $Cu-N_x$ containing carbon support for highly efficient electrocatalytic hydrogen evolution. *Appl. Surf. Sci.* **2022,** *591*, 153057.
(S18) Li, Y.; Jiang, K. Y.; Yang, J.; Zheng, Y. Y.; Hübner, R.; Ou, Z. W.; Dong, X.; He, L. Q.; Wang, H. L.; Li, J.; Sun, Y. J.; Lu, X. B.; Zhuang, X. D.; Zheng, Z. K.; Liu, W., Tungsten Oxide/Reduced Graphene Oxide Aerogel with Low-Content Platinum as High-Performance Electrocatalyst for Hydrogen Evolution Reaction. *Small* **2021,** *17*, 2102159.
(S19) Ramesh, R.; Han, S.; Nandi, D. K.; Sawant, S. Y.; Kim, D. H.; Cheon, T.; Cho, M. H.; Harada, R.; Shigetomi, T.; Suzuki, K.; Kim, S. H., Ultralow Loading (Single-Atom and Clusters) of the Pt Catalyst by Atomic Layer Deposition Using Dimethyl ((3,4-η) *N*,*N*-dimethyl-3-butene-1-amine-*N*) Platinum (DDAP) on the High-Surface-Area Substrate for Hydrogen Evolution Reaction. *Adv. Mater. Interfaces* **2021,** *8*, 2001508.
(S20) Yang, Q.; Liu, H. X.; Yuan, P.; Jia, Y.; Zhuang, L. Z.; Zhang, H. W.; Yan, X. C.; Liu, G. H.; Zhao, Y. F.; Liu, J. Z.; Wei, S. Q.; Song, L.; Wu, Q. L.; Ge, B. Q.; Zhang, L. Z.; Wang, K.; Wang, X.; Chang, C. R.; Yao, X. D., Single Carbon Vacancy Traps Atomic Platinum for Hydrogen Evolution Catalysis. *J. Am. Chem. Soc.* **2022,** *144*, 2171-2178.
(S21) Chen, Z. J.; Cao, G. X.; Gan, L. Y.; Dai, H.; Xu, N.; Zang, M. J.; Dai, H. B.; Wu, H.; Wang, P., Highly Dispersed Platinum on Honeycomb-like NiO@Ni Film as a Synergistic Electrocatalyst for the Hydrogen Evolution Reaction. *ACS Catal.* **2018,** *8*, 8866-8872.
(S22) Wang, Z. Y.; Yang, J.; Gan, J.; Chen, W. X.; Zhou, F. Y.; Zhou, X.; Yu, Z. Q.; Zhu, J. F.; Duan, X. Z.; Wu, Y. E., Electrochemical conversion of bulk platinum into platinum single-atom sites for the hydrogen evolution reaction. *J. Mater. Chem. A* **2020,** *8*, 10755-10760.
(S23) Gao, J. J.; Du, P.; Zhang, Q. H.; Shen, X.; Chiang, F. K.; Wen, Y. R.; Lin, X.; Liu, X. J.; Qiu, H. J., Platinum single atoms/clusters stabilized in transition metal oxides for enhanced electrocatalysis. *Electrochim. Acta* **2019,** *297*, 155-162.

(S24) Shi, Y.; Ma, Z. R.; Xiao, Y. Y.; Yin, Y. C.; Huang, W. M.; Huang, Z. C.; Zheng, Y. Z.; Mu, F. Y.; Huang, R.; Shi, G. Y.; Sun, Y. Y.; Xia, X. H.; Chen, W., Electronic metal-support interaction modulates single-atom platinum catalysis for hydrogen evolution reaction. *Nat. Commun.* **2021,** *12*, 3021.

(S25) Cheng, X.; Li, Y. H.; Zheng, L. R.; Yan, Y.; Zhang, Y. F.; Chen, G.; Sun, S. R.; Zhang, J. J., Highly active, stable oxidized platinum clusters as electrocatalysts for the hydrogen evolution reaction. *Energy Environ. Sci.* **2017,** *10*, 2450-2458.

(S26) Wang, M. M.; Wang, M. J.; Zhan, C. H.; Geng, H. B.; Li, Y. H.; Huang, X. Q.; Bu, L. Z., Ultrafine platinum-iridium distorted nanowires as robust catalysts toward bifunctional hydrogen catalysis. *J. Mater. Chem. A* **2022,** *10*, 18972-18977.

(S27) Wu, Y. C.; Wei, W.; Yu, R. H.; Xia, L. X.; Hong, X. F.; Zhu, J. X.; Li, J. T.; Lv, L.; Chen, W.; Zhao, Y.; Zhou, L.; Mai, L. Q., Anchoring Sub-Nanometer Pt Clusters on Crumpled Paper-Like MXene Enables High Hydrogen Evolution Mass Activity. *Adv. Funct. Mater.* **2022,** *32*, 2110910.
